# Energy-efficient integrated silicon optical phased array

**DOI:** 10.1007/s12200-023-00076-1

**Published:** 2023-09-22

**Authors:** Huaqing Qiu, Yong Liu, Xiansong Meng, Xiaowei Guan, Yunhong Ding, Hao Hu

**Affiliations:** 1 DTU Electro, Department of Electrical and Photonics Engineering, Technical University of Denmark, Kgs. Lyngby, DK-2800 Denmark; 2 Interuniversity Microelectronics Center (IMEC), Kapeldreef 75, Leuven, 3001 Belgium; 3 Jiaxing Key Laboratory of Photonic Sensing and Intelligent Imaging, Jiaxing, 314000 China; 4 Intelligent Optics and Photonics Research Center, Jiaxing Research Institute, Zhejiang University, Jiaxing, 314000 China

**Keywords:** Optical phased array, Optical phase shifter, Silicon photonics, Integrated optics

## Abstract

An optical phased array (OPA) is a promising non-mechanical technique for beam steering in solid-state light detection and ranging systems. The performance of the OPA largely depends on the phase shifter, which affects power consumption, insertion loss, modulation speed, and footprint. However, for a thermo-optic phase shifter, achieving good performance in all aspects is challenging due to trade-offs among these aspects. In this work, we propose and demonstrate two types of energy-efficient optical phase shifters that overcome these trade-offs and achieve a well-balanced performance in all aspects. Additionally, the proposed round-spiral phase shifter is robust in fabrication and fully compatible with deep ultraviolet (DUV) processes, making it an ideal building block for large-scale photonic integrated circuits (PICs). Using the high-performance phase shifter, we propose a periodic OPA with low power consumption, whose maximum electric power consumption within the field of view is only 0.33 W. Moreover, we designed Gaussian power distribution in both the azimuthal ($\varphi$) and polar ($\theta$) directions and experimentally achieved a large sidelobe suppression ratio of 15.1 and 25 dB, respectively.

**Graphical Abstract:**

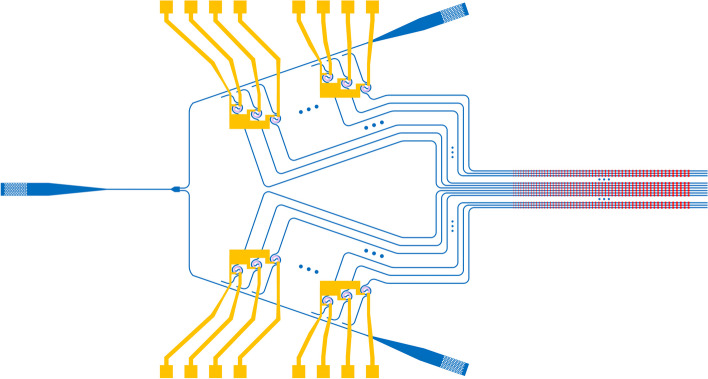

## Introduction

In the past few decades, the invention of radio detection and ranging (Radar) [1] has brought tremendous convenience to people’s lives, with its wide applications in socioeconomic development such as meteorological forecasting, resource exploration, and environmental monitoring, as well as in scientific research areas such as astrophysics, atmospheric physics, and ionospheric structure research. The working principle of radar sensors, including ultrasonic and millimeter-wave radar, involves launching an ultrasonic wave or millimeter-wave towards a target, followed by the comparison of the received echo reflected from the target with the transmitted signal. Through calculation and analysis, relevant information about the target such as distance, azimuth, altitude, speed, and shape can be obtained. Radar sensors have already achieved maturity and are extensively utilized in various industries. The primary limitations of radar sensors are their restricted detection range and spatial resolution. For instance, The detection range of the millimeter-wave radar is directly constrained by frequency band losses, which necessitates the use of higher frequency bands to achieve longer detection distances. Moreover, millimeter-wave radar cannot perceive pedestrians and is unable to accurately model the surrounding obstacles.

The concept of light detection and ranging (LiDAR) systems was first proposed in the 1960s, leveraging their short wavelength, high resolution, good directionality, and electromagnetic immunity [2–7]. As a dynamic distance measurement method, LiDAR has garnered considerable attention in both academia and industry. Due to their different working principles, LiDAR can be classified into several categories [8]. Based on whether the laser beam is scanned or not, LiDAR can be categorized into two types: non-scanning LiDAR and scanning LiDAR. Flash LiDAR is a representative example of non-scanning LiDAR [9, 10]. For scanning LiDAR, it can be further categorized as either mechanical or non-mechanical scanning LiDAR, depending on the type of scanning method used. Both micro-electro-mechanical system (MEMS) LiDAR [11, 12] and motorized mechanical LiDAR are classified as mechanical scanning LiDAR [13], as they employ mechanical scanning components. Conversely, a non-mechanical scanning LiDAR system typically utilizes an optical phased array (OPA) [14–17] for beam steering, without any mechanical parts in the system. Meanwhile, MEMS LiDAR is also known as quasi-solid-state LiDAR, as its moving components are solely employed for laser beam scanning and not for moving any optical components. Flash LiDAR and OPA LiDAR are also called solid-state LiDAR, as they do not have any moving parts within the system.

The OPA LiDAR, in comparison to other LiDARs, is a fully integrated solid-state scanning solution that offers several key advantages, including reduced device size and cost, increased lifespan, and simplified mass production. Additionally, the OPA LiDAR employs electrical signals for beamforming and beam steering, which allows for a reduction in the number of lasers required in the emitter module and lowers overall cost. Given these benefits, OPA technology is widely considered to be the optimal solution for producing low-cost, mass-produced LiDAR devices.

**Fig. 1:**
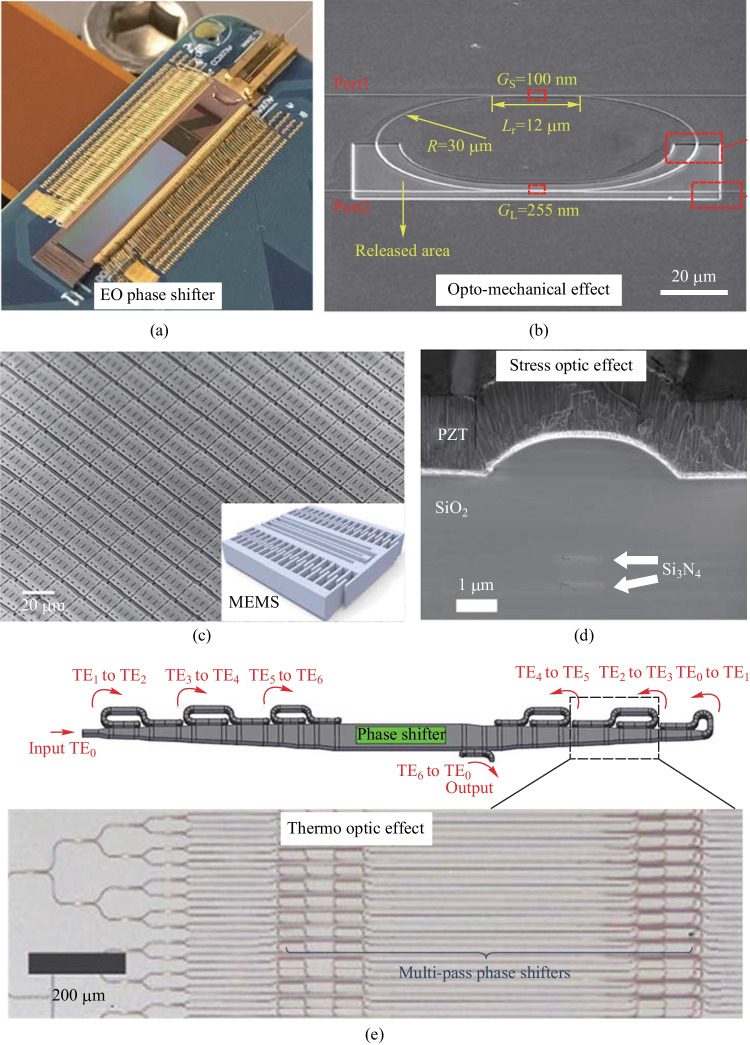
**a** A 512-channel OPA example of the electro-optic phase shifter [18]. **b** The optomechanical effect-based phase shifter [19]. **c** A 160 $\times$ 160 2D-OPA utilizing MEMS. **d** The stress-optic effect (PZT) based phase shifter. **e** A 512-channel OPA scheme based on the multipass thermo-optic effect [20]

**Table 1:** Performance comparison of examples from different optical phase shifters

Type	*P*$_\uppi$	Voltage (2$\uppi$)	Modulation speed	Loss /dB	Footprint /($\upmu {\textrm{m}}$ $\times$ $\upmu {\textrm{m}}$)	Stability	Fabrication difficulty
EO forward [21]	1.7 mW	1.1 V	20 MHz	3.2	2.5 $\times$ 1000	Good	Medium
EO inverse [22]	1 $\upmu {\textrm{W}}$	–16 V	25.6 GHz	4.2	2 $\times$ 4000	Good	Medium
MEMS [23]	2.7 nW	10.5 V	55 kHz	—	16.5 $\times$ 14	Bad	Hhigh
PZT [24]	0.16 $\upmu {\textrm{W}}$	16 V	94 kHz	5	1027 $\times$ 1027	Good	High
TO [25]	2.56 mW	1.1 V	10.1 kHz	1.23	109 $\times$ 21	Good	Easy

Over the past decade, there has been significant progress in photonic integration. A photonic integrated circuit (PIC) enables the integration of multiple components on a single chip, including lasers, modulators, optical signal processors, optical amplifiers, and photodetectors, which are essential for the development of integrated OPAs. Micro- and nano-fabrication techniques, widely used in the microelectronics industry, have been utilized for fabricating PICs, leading to robust, reproducible, and high-quality photonic integration platforms [17]. Compared with bulk optical components, PICs usually employ electrical signals to control the chip, which eliminates the need for mechanical movement. Additionally, PICs can provide large bandwidth, well into the gigahertz range, further contributing to their suitability for OPAs. Furthermore, the micro- and nano-fabrication processes can guarantee uniform components for each channel, which is critical for avoiding phase errors, given that OPAs are highly sensitive to such errors.

Recently, integrated OPAs have garnered significant research interest [26–39]. Many researchers have demonstrated integrated OPAs on various platforms, such as silicon-on-insulator (SOI) [28, 29, 31, 33–35, 37, 39], silicon nitride ($\mathrm {Si_3N_4}$) [26, 27, 31], and indium phosphide (InP) [40, 41]. Moreover, attempts at hybrid integration of OPA have also been made to combine the strengths of different platforms [36, 38].

One of the critical components of an OPA is the optical phase shifter, which plays a vital role in determining the device’s power consumption, modulation speed, and insertion loss. The power consumption can limit the scalability of the OPA and ultimately affect the beamwidth, which determines the spatial resolution in the far field. The modulation speed is a critical factor that impacts the frame rate of the 3D point cloud generated by the OPA when applied to Lidar. The insertion loss of the optical phase shifter influences the output power of the OPA, thereby determining the maximum ranging distance of a Lidar system. Currently, the farthest ranging distance achieved with OPA Lidar is reported to be 50 m [42].

Based on various principles, optical phase shifters can be classified into several categories, including electro-optic (EO) effect [18], optomechanical effect [19], MEMS [23], stress-optic effect [43], and thermo-optic (TO) effect [20, 44–46].

The principle of the EO phase shifter is based on the free carrier dispersion effects, where by regulating the carrier concentration in the waveguide, the refractive index is altered to achieve phase modulation. While the modulation speed is high, reaching tens of megahertz (using carrier injection modulation with forward bias [21]) and even tens of gigahertz (using carrier depletion modulation with inverse bias [22]), there are notable drawbacks, such as mm-long device, large insertion loss, and high applied voltage, particularly for the carrier depletion type, which typically requires tens of volts. Additionally, the primary drawback of applying EO phase shifters to OPA is that the intensity changes when the phase is modulated, which can be detrimental to the OPA’s stability. In 2018, Christopher V. Poulton et al. demonstrated a 512-channel 1D-OPA with forward-biased EO phase shifters and a time constant of 10 ns, corresponding to a modulation speed of 32 MHz. The power consumption is as small as 2 $\upmu {\textrm{W}}$/${\uppi }$, as is shown in Fig. 1a [18].

The optomechanical effect relies on the optical gradient force to deform the waveguide and bring about a corresponding change in the refractive index. Despite its low power consumption, the optomechanical approach suffers from a relatively sluggish modulation speed, and the device is prone to instability [19]. An optomechanical effect-based phase shifter is shown in Fig. 1b.

The MEMS phase shifter operates by applying a gradient electric field to deform the waveguide and thus modulate the mode effective refractive index. This technique boasts impressive performance in terms of power consumption and insertion loss. The modulation speed is typically limited by the mechanical frequency, but it can still reach tens of kilohertz. In 2019, Youmin Wang et al. utilize a novel MEMS phase shifter to demonstrate a 160 $\times$ 160 2D-OPA, as depicted in Fig. 1c. The power consumption of the MEMS phase shifter is only 2.7 nW (at 12 V driving voltage), and the modulation speed is 55 kHz [23].

The stress-optic effect is a novel approach to phase modulation in integrated optics. PZT piezoelectric ceramics, which exhibit an inverse piezoelectric effect, can generate a micro-displacement corresponding to a voltage signal under an applied voltage. This technique offers nanometer-level resolution and microsecond-level response speed, which is highly desirable for achieving precise optical phase modulation. While the fabrication is extremely hard and the technique is immature. The cross-section of the PZT on a $\mathrm {Si_3N_4/Si}$ waveguide is shown in Fig. 1d.

The thermo-optic effect relies on heating the waveguide to alter the effective refractive index. While the TO phase shifter boasts negligible insertion loss and a small footprint, it is somewhat limited in terms of power consumption and modulation speed, typically reaching only tens of kilohertz. Nonetheless, it remains the most widely utilized mechanism due to its straightforward principle and easy fabrication process. In a recent research by Steven A. Miller et al., a novel TO phase shifter was utilized in a 512-channel OPA. The researchers demonstrated a multi-pass structure optical phase shifter [20] that employed a series of mode converters to ensure that light traveled back and forth seven times within the same waveguide, effectively reducing power consumption, as is shown in Fig. 1e. However, this approach resulted in increased insertion loss due to the presence of mode converters, and the structure exhibited poor robustness.

We provide a comparison of several examples belonging to different categories of optical phase shifters. As demonstrated in Table 1, the TO-based phase shifter exhibits a well-balanced performance across all parameters.

## Energy-efficient optical phase shifter

### Introduction to thermo-optic phase shifter

The underlying principle behind the micro-heater is to utilize the thermo-optic effect, which involves the modulation of the effective refractive index (*n*) of a silicon waveguide by heating it using a micro-heater. Specifically, the micro-heater generates heat, which increases the temperature of the silicon waveguide. This, in turn, leads to a change in the effective refractive index of the waveguide, resulting in a phase shift of the light propagating through it. The rate of change of *n* with respect to temperature (*T*) is defined as the thermo-optic coefficient.

**Fig. 2:**
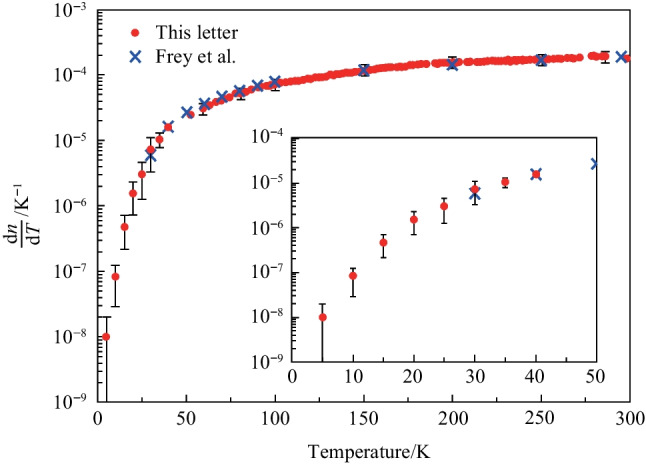
The thermo-optic coefficient in silicon waveguide from 5 to 300 K (room temperature) [47, 48]

To quantify this effect, the thermo-optic coefficient of a silicon waveguide was measured over a temperature range of 5 to 300 K, as illustrated in Fig. 2. At room temperature (300 K), which is a critical operating condition, the thermo-optic coefficient was found to be

1 $$\begin{aligned} \dfrac{{\text{d}}n}{{\text{d}}T} = 1.8 \times 10^{-4} \ {\text{K}}^{-1}. \end{aligned}$$

The red dot in Fig. 2 represents the experimental result reported by J. Komma et al. [47], while the blue cross corresponds to the experimental result obtained by Frey et al. [48]. The data in Fig. 2 show that the thermo-optic coefficient for the silicon waveguide is approximately constant when the temperature exceeds 200 K, with a value of approximately 1.8 $\times$ $10^{-4}$ K$^{-1}$. This finding is supported by the experimental results of both research groups.

However, the high power consumption of typical TO phase shifters, e.g., approximately 24.4 mW/$\uppi$ [25], has become a significant obstacle to large-scale integration, which can lead to a significant increase in temperature and cause thermal crosstalk or even damage to the chip.

**Fig. 3:**
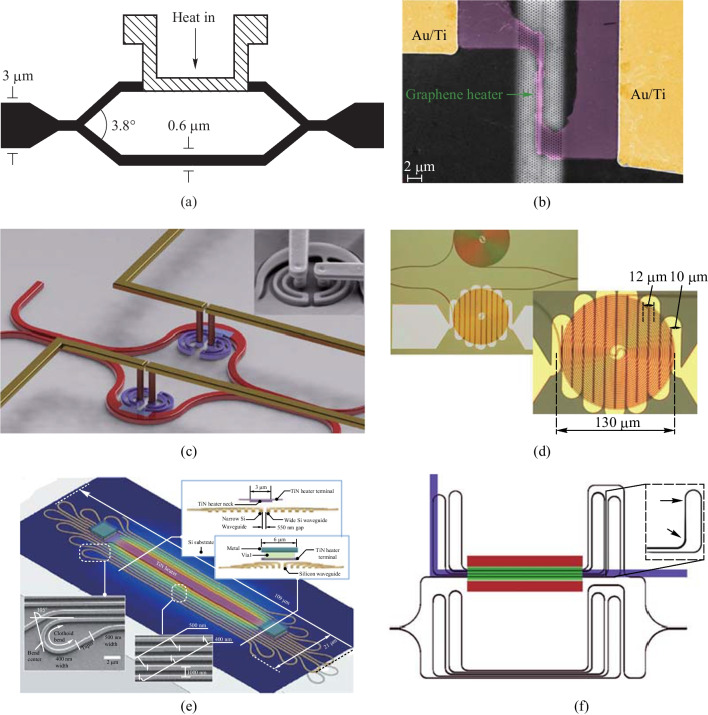
**a** A typical TO phase shifter [49]. **b** A Slow-light-enhanced graphene microheater on silicon PhCWs [51]. **c** A compact doped silicon phase shifter [45]. **d** A folded-waveguide phase shifter [52]. **e** A dense dissimilar waveguide routing phase shifter [53]. **f** A Clothoid bends based well-balanced phase shifter [25]

Another typical TO phase shifter without any optimization was demonstrated by R. L. Espinolaet al., which consumed 50 mW/$\uppi$ with a micro-heater [49], as is shown in Fig. 3a. Later, various methods have been proposed to reduce the power consumption of silicon TO phase shifters. Siqi Yan et al. proposed a slow-light-enhanced graphene micro-heater on silicon photonic crystal waveguides [50, 51], which consumed as little as 2 mW/$\uppi$ owing to the graphene’s direct contact with the waveguide [51]. The structure is illustrated in Fig. 3b. However, this method suffered from significant insertion loss due to optical absorption from graphene.

Other methods to reduce power consumption have also been explored. Michael R. Watts et al. optimized the optical loss in a tiny radius bend and used lightly *n*-type doped bent waveguides as a resistor and heavily *n*$^+$-doped silicon leads as conductors to achieve a power consumption of 12.7 mW/$\uppi$ with a footprint of only 6 $\upmu {\textrm{m}}$ $\times$ 6 $\upmu {\textrm{m}}$ [45], which is shown in Fig. 3c. The doped Si waveguide leverages the waveguide material as the resistive element, enabling it to exhibit excellent performance in terms of modulation speed and compact size. Nevertheless, this approach is accompanied by notable insertion loss and offers limited potential for reducing power consumption, mainly attributed to the doping process involved. Adam Densmore et al. used a folded silicon waveguide and a folded micro-heater to achieve a low power consumption of 6.5 mW/$\uppi$ (Fig. 3d), with the heat generated by the heater being absorbed by the waveguide, including the residual heat [52]. Kyle Murray et al. demonstrated a TO-based shifter with a similar principle to that of Densmore et al. with a power consumption of only 3.8 mW/$\uppi$ but a footprint of 800 $\upmu {\textrm{m}}$ $\times$ 180 $\upmu {\textrm{m}}$ [53].

In 2019, Sungwon Chung et al. proposed a TO-based phase shifter using Clothoid bends under a commercial silicon photonic fabrication process [25], the schematic is shown in Fig. 3e. This method achieved a well-balanced performance, with power consumption, modulation speed, insertion loss, and footprint of 2.56 mW/$\uppi$, 10.1 kHz, 1.23 dB, and 109 $\upmu {\textrm{m}}$ $\times$ 21 $\upmu {\textrm{m}}$, respectively. However, the design is complex, especially for the Clothoid bends design.

In addition, there exist other techniques for enhancing the TO efficiency, such as incorporating air trenches around the phase shifter or undercutting the Si substrate below it [54, 55], as is shown in Fig. 3f. Due to the weak thermal conductivity of SiO$_2$ and air in comparison to Si, these methods result in strong heat confinement.

In general, there are several trade-offs among the four main aspects of a TO phase shifter, which include power consumption, insertion loss, modulation speed, and footprint. It is a challenge to break through the trade-offs and achieve good performance in all aspects simultaneously.

### Racetrack-spiral silicon phase shifter

We propose a solution to the issue of high power consumption in conventional TO phase shifters, while also addressing the need for balanced performance in terms of insertion loss, modulation speed, and footprint. To achieve this, we introduce an energy-efficient racetrack-spiral silicon phase shifter and provide experimental evidence of its well-balanced performance.

**Fig. 4:**
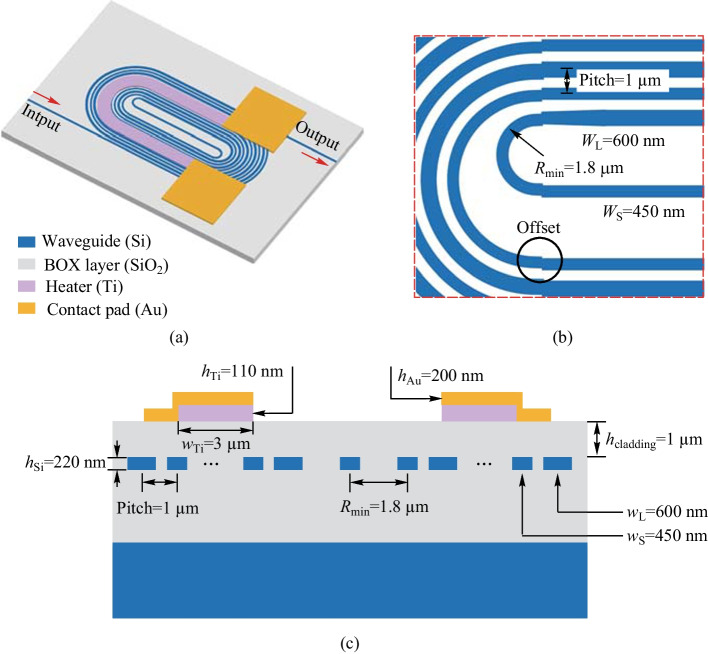
**a** Schematic of the proposed silicon TO racetrack-spiral phase shifter using a spiral waveguide. **b** The zoomed-in figure of the offset part [56]. **c** Schematic of the cross-section with the well-designed parameters

The proposed T/O racetrack-spiral silicon optical phase shifter is illustrated in Fig. 4a. The device is composed of a densely packed silicon spiral waveguide and a wide Titanium (Ti) heater, which is responsible for adjusting the effective refractive index of the waveguide. Due to the high density of the waveguide, the heat generated by the Ti heater is effectively absorbed by the underlying spiral structure, leading to a significant reduction in power consumption required for a $\uppi$ phase shift. Moreover, the device features a compact footprint, which can be attributed to the reduction of blank space in the structure and the central symmetry design.

The spiral waveguide features a carefully designed series of bend connectors and straight waveguides. The pitch of the straight waveguides has been optimized to only 1 $\upmu {\textrm{m}}$ to minimize the footprint, while the pitches between the bend connectors vary due to individually tailored structures. Two adjacent straight waveguides have a large difference in width, with $w_\text{L}$ = 600 nm and $w_\text{S}$ = 450 nm, to achieve a large phase mismatch and avoid coupling between them, as illustrated in the zoomed-in Fig. 4b. The straight waveguides are connected by a series of bend connectors, which have a total length of 1.2 mm and a minimum radius of only 1.8 $\upmu {\textrm{m}}$. Each bend connector has a different radius, which is carefully designed to minimize the bend loss and the overall footprint of the phase shifter. Most bend connectors connect two straight waveguides with the same waveguide width ($w_\text{L}$ or $w_\text{S}$), except for the two bend connectors with the smallest radius, which connect two waveguides with different widths ($w_\text{L}$ and $w_\text{S}$). An adiabatic taper is placed between the smallest connector and the corresponding waveguide to reduce mode mismatch loss due to different waveguide widths. Moreover, the offsets between different bend connectors and straight waveguides are individually optimized to further reduce mode mismatch loss caused by sharp bending. It should be noted that for all the other bend connectors, the bend width is the same as the corresponding straight waveguide, and the mode mismatch loss is caused by sharp bending instead of waveguide width differences.

Figure 4c displays a cross-sectional diagram featuring well-optimized parameters. The silicon-on-insulator (SOI) substrate was procured with a top silicon layer thickness of 220 nm, coupled with a buried oxide layer (BOX) that has a thickness of 2 $\upmu {\textrm{m}}$. To construct the racetrack-spiral phase sifter, the silicon waveguide is first patterned using E-beam lithography (EBL) and then fully etched through deep reactive ion etching (DRIE). To protect the silicon waveguide and minimize optical losses due to subsequent metal layer deposition, a 1 $\upmu {\textrm{m}}$ thick layer of silicon dioxide (SiO$_2$) is deposited on the waveguide using plasma-enhanced chemical vapor deposition (PECVD). It is worth noting that the choice of a 1 $\upmu {\textrm{m}}$ SiO$_2$ cladding is a trade-off between the absorption loss due to the metal layer and the phase shifter efficiency. A thicker cladding would reduce the absorption loss but decrease the efficiency of the phase shifter. Then the Ti heater layer and the Au conductor layer are deposited using the lift-off process to construct the micro-heater on top of the racetrack-spiral waveguide.

**Fig. 5:**
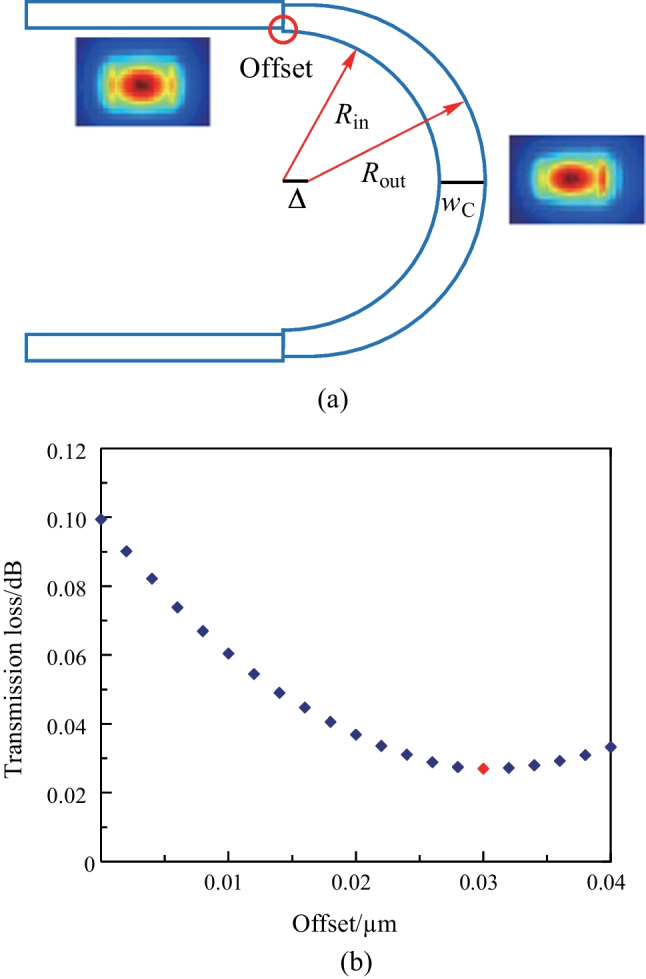
**a** Schematic of transmission loss optimization for a bent connector. **b** Mode mismatch loss versus offset optimization

The illustration presented in Fig. 5 provides insight into the optimization process for minimizing the loss of bend connectors. The transmission loss of a bent connector is comprised of several factors, including bend loss, mode mismatch loss, and loss from higher-order modes that may be excited in widened bends. Notably, when waveguide width remains constant, the primary source of mode mismatch loss is associated with sharp bends. Figure 5a depicts two distinct mode profiles. The left profile pertains to a straight waveguide, exhibiting a symmetric mode profile. In contrast, the mode profile of the sharp bend waveguide (right profile) deviates significantly from the center to the edge. This deviation becomes more pronounced when a sharp bend waveguide is directly connected to a straight waveguide of equal width. The misalignment of the two-mode profiles results in a significant mode mismatch loss during transmission.

**Fig. 6:**
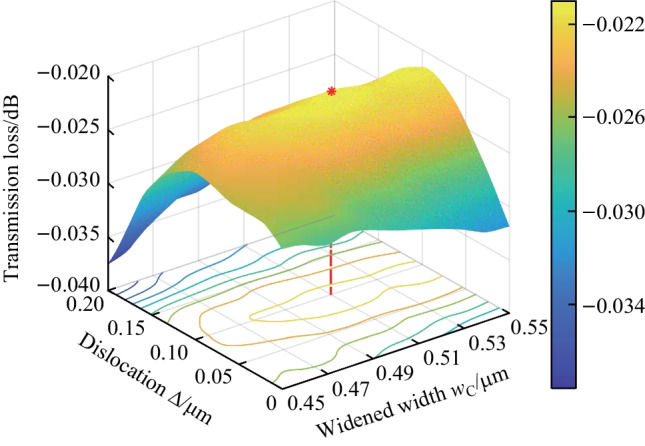
Bend loss and higher-order mode loss optimization with dislocation $\Delta$ and widened width $w_\text{C}$

To effectively reduce the mode mismatch loss, it is recommended to introduce an offset between the sharp bend connector and the straight waveguide. This offset realigns the center of the two-mode profiles, mitigating the loss. However, to minimize the footprint of the phase shifter, it is preferred to have a small bend radius for all bend connectors. As such, bend loss dominates the total loss and cannot be ignored. To address this issue, a dislocation $\Delta$ is introduced between the center of the outer boundary ($R_\text{out}$) and the inner boundary ($R_\text{in}$). This results in a widened waveguide width in the bend arc, which reduces the bend loss, but increases the higher-order mode loss due to the wider waveguide width. To mitigate the higher-order mode loss, the bent waist ($w_\text{C}$) is optimized in conjunction with the dislocation. In this manner, the outer boundary ($R_\text{out}$) takes on a half-circular curve, while the inner boundary ($R_\text{in}$) behaves like a half-ellipse curve.

**Fig. 7:**
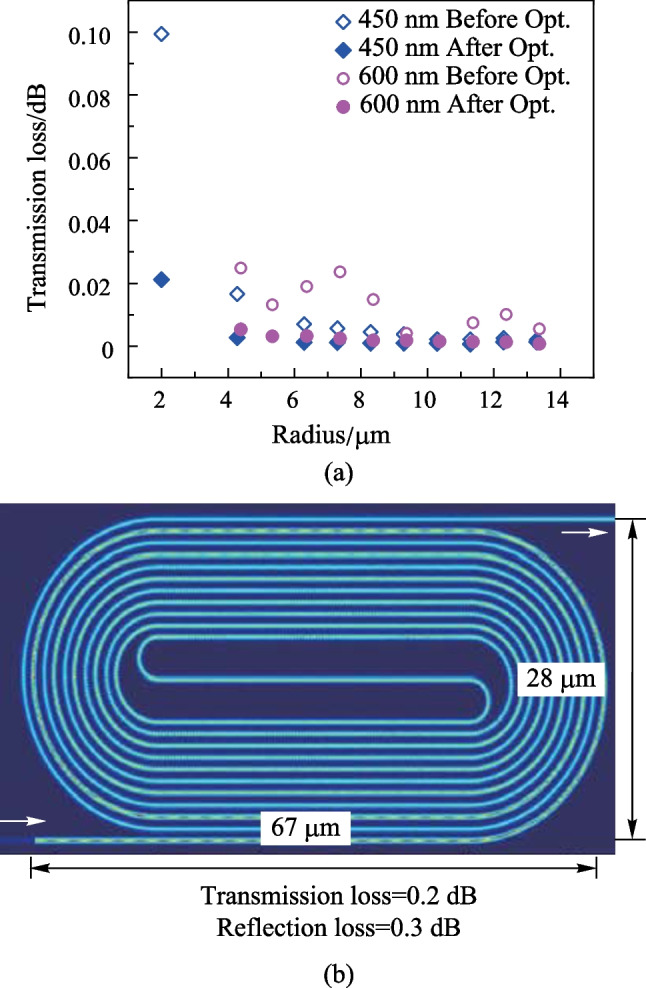
**a** Transmission loss for a bent connector versus bend radius before and after the optimization for $w_\text{S}$ = 450 nm (blue) and $w_\text{L}$ = 600 nm (purple). **b** Simulated light propagation with calculated total transmission loss and total reflection loss

We initiated the design process by selecting the parameters $w_\text{S}$ = 450 nm, $R_\text{in}$ = 1.55 $\upmu {\textrm{m}}$, and $R_\text{out}$ = 2 $\upmu {\textrm{m}}$, which correspond to an effective radius of $R_\text{eff}$ = 1.8 $\upmu {\textrm{m}}$, for the smallest bend connector. We then used the finite-difference-time-domain (FDTD) method to simulate and analyze the transmission loss of the smallest bend connector as a function of the offset. The results of the optimization are presented in Fig. 5b. It can be observed that the mode mismatch loss is minimized when the offset is 30 nm (represented by the red rhombus in Fig. 5b) and is 3.7 times lower than that without the offset. We then fixed the offset as 30 nm and conducted simulations of the bend loss and higher-order mode loss simultaneously. These losses are determined by two variables, namely, the dislocation $\Delta$ between the center of $R_\text{in}$ and $R_\text{out}$, and the widened bend waist width $w_\text{C}$. The results of the simulations are shown in Fig. 6. The minimal loss is obtained when the dislocation $\Delta$ = 0.1 $\upmu {\textrm{m}}$ and the widened width $w_\text{C}$ = 510 nm, which is approximately 1.3 times lower than the case of $\Delta$ = 0 $\upmu {\textrm{m}}$, $w_\text{C}$ = 450 nm, and offset = 30 nm. The total transmission loss for the case of $w_\text{S}$ = 450 nm and $R_\text{eff}$ = 1.8 $\upmu {\textrm{m}}$ is approximately 0.02 dB with all the optimization processes (i.e., $\Delta$ = 0.1 $\upmu {\textrm{m}}$, $w_\text{C}$ = 510 nm, and offset = 30 nm), which is approximately 5 times lower than that (approximately 0.1 dB) of using the simple half-circular bend connector without any optimization (i.e., $\Delta$ = 0 $\upmu {\textrm{m}}$, $w_\text{C}$ = 450 nm, and offset = 0 nm).

Using a loss optimization method, we performed simulations to optimize the transmission loss for different radii of bend connectors with widths of 450 nm and 600 nm, as depicted in Fig. 7a. Additionally, we determined that the optimized waveguide offset varied with the radius. To evaluate the effectiveness of the optimization, we also simulated the bend connectors without loss optimization as references. The mathematical transmission loss of the racetrack-spiral phase shifter, after loss optimization, was found to be 0.05 dB, which was approximately five times lower than the loss of 0.26 dB for the bend connectors without optimization. Next, we connected the individually optimized bend connectors with straight waveguides to construct the phase shifter and analyzed its insertion loss. To do this, we imported the entire phase shifter structure into ANSYS’s Lumerical FDTD Solutions, where we simulated the light propagation through the phase shifter as shown in Fig. 7b. The phase shifter footprint measured 67 $\upmu {\textrm{m}}$ $\times$ 28 $\upmu {\textrm{m}}$, and the total transmission loss was calculated as 0.2 dB, which was higher than the aggregate transmission loss of 0.05 dB due to the reconstruction of the structure and coupling loss. We also simulated a total reflection loss of 0.3 dB resulting from the offset and coupling in the reverse direction.

**Fig. 8:**
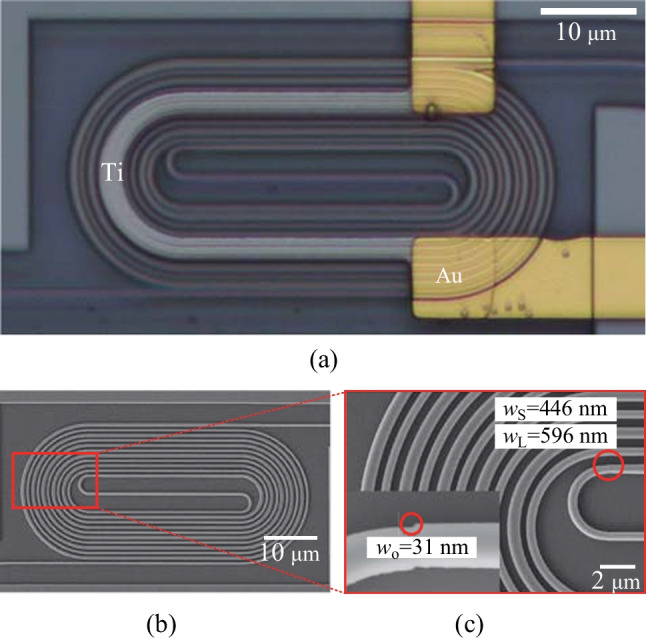
**a** Microscope image of the racetrack-spiral phase shifter. **b** SEM image of the phase shifter. **c** Zoomed-in SEM image of the offset part, the measured 31 nm matches well with the design of 30 nm

In Fig. 8a, a microscope image of the racetrack-spiral phase shifter is presented, where the Au connect line is shown in gold color and the Ti heater is depicted as a gray bent part on top of the Si waveguide. Prior to depositing SiO$_2$, the feature size of the device is measured using a scanning electron microscope (SEM), as illustrated in Fig. 8b, with a zoom-in figure shown in Fig. 8c. The measured values of $w_\text{L}$ and $w_\text{S}$ are found to be 596 and 446 nm, respectively, which match the design parameters. Moreover, the left-down inset in Fig. 8c reveals an offset ($w_\text{O}$) of 31 nm, demonstrating the successful fabrication of the 30 nm offset using EBL.

Before depositing the Ti heater on the chip, we conducted a resistance test with various Ti heater widths and lengths. It is crucial to ensure that the resistance of the Ti heater is at an appropriate value. If the resistance is too high, the voltage required to achieve a 2$\uppi$ phase shift may exceed the output range of the Field Programmable Gate Array (FPGA), which is fixed to 3.3 V. While other integrated circuit solutions could generate voltage outputs exceed 3.3 V, they are complex and unnecessary. Therefore, we designed the width, thickness, and height of the Ti heater to be 3 $\upmu {\textrm{m}}$, 80 $\upmu {\textrm{m}}$, and 110 nm, respectively, resulting in a resistance of $\sim$ 330 $\Omega$. The thickness of the Aurum (Au) conductor should be greater than that of Ti to ensure that the Au layer on top of and beside the Ti layer is electrically connected. We designed the thickness of the Au layer to be 200 nm for this purpose. However, for the round-spiral phase shifters and optical phase amplifiers (OPAs) introduced in subsequent sections and chapters, we increased the thickness of the Au layer to 500 nm to decrease the resistance between the FPGA and the Ti heater, save energy, and minimize phase crosstalk.

**Fig. 9:**
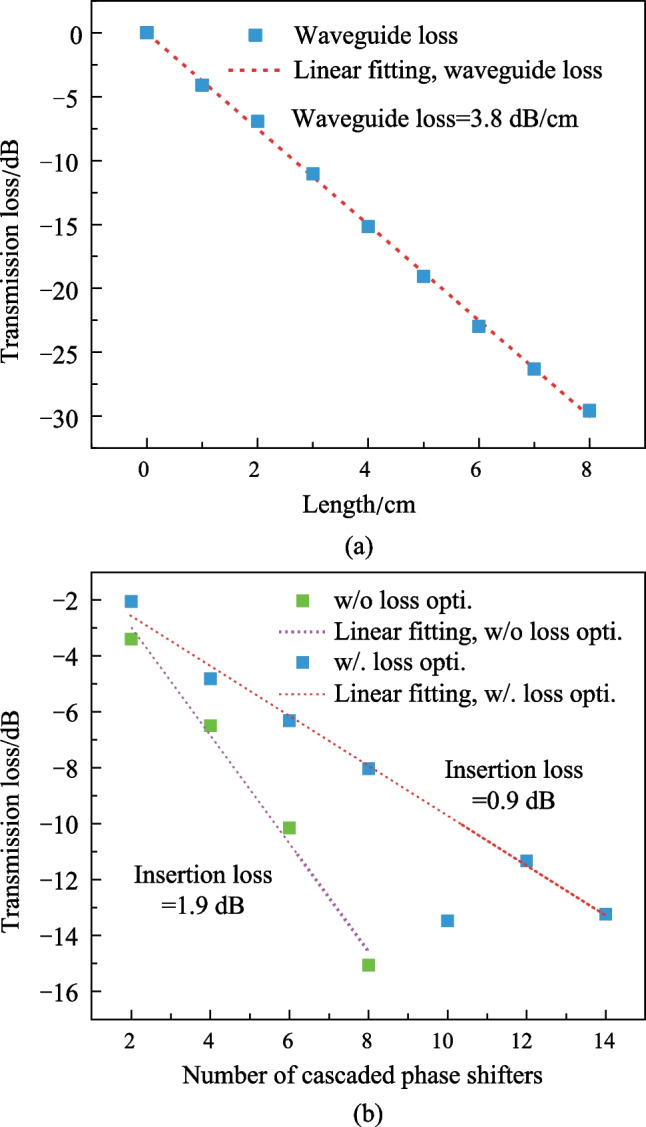
**a** Meausred waveguide loss of 450-nm-width silicon straight waveguide. **b** Measured insertion loss of the racetrack-spiral phase shifter with and without loss optimization

We have fabricated straight waveguides on the same chip as the proposed racetrack-spiral waveguide, with a width of 450 nm and varying lengths from 1 to 8 cm in 1 cm increments. Using the cutback method, we measured the waveguide loss to be 3.8 dB/cm, as shown in Fig. 9a. We also fabricated reference phase shifters on the same chip without any loss optimization but keeping all other parameters the same. The loss measurements for the phase shifters with and without loss optimization are shown in Fig. 9b, where the insertion loss decreases from 1.9 dB (without loss optimization) to 0.9 dB (with loss optimization). We should note that the insertion loss for the device with 10 cascaded phase shifters is abnormally high due to unintended particles on that device. The measured total insertion loss of the fabricated racetrack-spiral phase shifter, which includes bend loss (0.2 dB, simulated), reflection loss (0.3 dB, simulated), and waveguide loss (0.46 dB, calculated using a 3.8 dB/cm straight waveguide loss and 1.2 mm length), agrees well with the simulated total loss result.

**Fig. 10:**
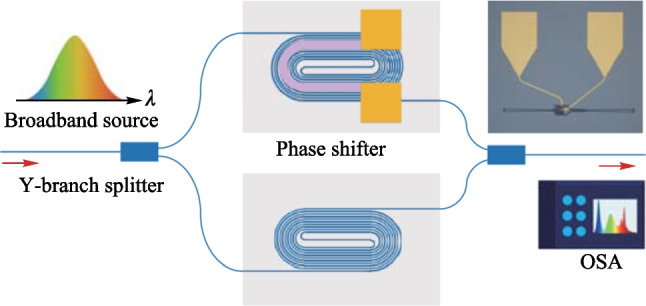
MZI structure for measuring the power consumption and the modulation speed

We devised a Mach-Zehnder interferometer (MZI) structure to quantify the power consumption of the proposed phase shifter, as presented in Fig. 10. To evaluate the optical spectrum of the MZI, we utilized the spontaneous emission from an Erbium-Doped Fiber Amplifier (EDFA) as a broadband input light source. The input light was introduced into the fabricated chip by a photonic crystal (PhC) vertical grating coupler, and then it was split into two paths by a compact Y-branch splitter. One path went through the phase shifter, whereas the other did not. Afterward, the light from both paths was combined by a Y-branch splitter, which induced interference. The output light was then detected by an optical spectrum analyzer (OSA). The integrated MZI structure is illustrated in the inset of Fig. 10.

**Fig. 11:**
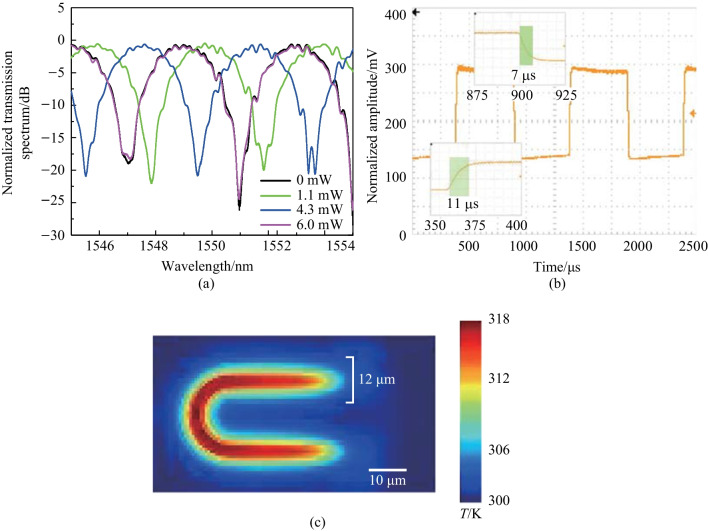
**a** Spectra of the MZI when electrical power varies from 0 mW to 6 mW, showing that the phase tuning efficiency is 3 mW/$\uppi$. **b** TO modulation speed of the phase shifter. The rising time and the falling time are 11 and 7 $\upmu {\textrm{s}}$, respectively, resulting in a modulation speed of 39 kHz. **c** Simulated temperature profile. The maximum temperature change is 18 K higher than the background temperature (300 K)

In order to achieve a high extinction ratio of the output spectrum, we introduced a spiral waveguide in the other arm of the MZI to balance the optical transmission loss. This waveguide has the same design as the racetrack-spiral phase shifter but with one less circle. The resistance of the metal heater on top of the silicon waveguide was measured to be 330 $\Omega$, with a width of 3 $\upmu {\textrm{m}}$, a length of 80 $\upmu {\textrm{m}}$, and a height of 110 nm. By increasing the voltage applied to the heater, the effective refractive index of the heated MZI arm also increased, causing a corresponding change in phase and interference wavelengths of the MZI. The experimental results presented in Fig. 11a show the MZI spectra as the applied electrical power varies from 0 mW to 6 mW, indicating a phase tuning efficiency of 3 mW/$\uppi$. The voltage needed to achieve a phase change of 2$\uppi$ is only 1.4 V, which can be supported by a commercial FPGA whose output voltage is typically 3.3 V.

To measure the modulation speed, we modulated the heater with a 1-kHz square-wave pulse train, as shown in Fig. 11b. The insets in Fig. 11b illustrate that the rising time (from 10% to 90%) and falling time (from 90% to 10%) are 11 and 7 $\upmu {\textrm{s}}$, respectively. Using the relationship between response time and cut-off frequency, the modulation speed was calculated to be 39 kHz.

In addition to experimental testing, we performed a simulation and analysis of the proposed racetrack-spiral phase shifter to investigate its temperature profile. We imported the device’s structure, in GDS file format, into ANSYS’s Lumerical DEVICE for heat analysis. To reflect practical conditions, we set the heater resistance in the simulation to be the same as the measured resistance of 330 $\Omega$. Applying a voltage of 1.4 V to the Au contacts generated heat in the Ti heater, causing the temperature of the Si waveguide to increase. As shown in Fig. 11c, the highest temperature at the center was 18 K above the background temperature (300 K). This ultra-low temperature change is significantly smaller than the $\sim$ 480 K temperature changes observed in previous phase shifters [45], making our device safe and long-lasting. The effective heating width was approximately 12 $\upmu {\textrm{m}}$, matching well with the device width (28 $\upmu {\textrm{m}}$), and any residual heat was efficiently absorbed by the densely distributed silicon spiral waveguide.

### Round-spiral silicon phase shifter

Despite achieving a good balance in power consumption (3 mW/$\uppi$), insertion loss (0.9 dB), modulation speed (39 kHz), and footprint (67 $\upmu {\textrm{m}}$ $\times$ 28 $\upmu {\textrm{m}}$), the proposed racetrack-spiral phase shifter has some drawbacks that must be addressed. One major issue is that the sub-micron offset between the bend connectors and the straight waveguides is not compatible with the standard commercial deep ultra-violet (DUV) processes with a minimum feature size of $\sim$ 150 nm. This limitation hinders large-scale integration and mass production, making it necessary to explore alternative fabrication methods such as electron beam lithography. Moreover, the offset between the bend connectors and the straight waveguides can cause significant reflection losses (0.3 dB in simulation compared to the 0.9 dB total experimental loss), which must be eliminated through better design. Finally, the complex design process of each bend connector requiring special and intricate shaping, presenting a challenge for scalability and cost-effectiveness. Therefore, further research is necessary to develop more streamlined and efficient design methods that can facilitate the large-scale fabrication of these devices.

**Fig. 12:**
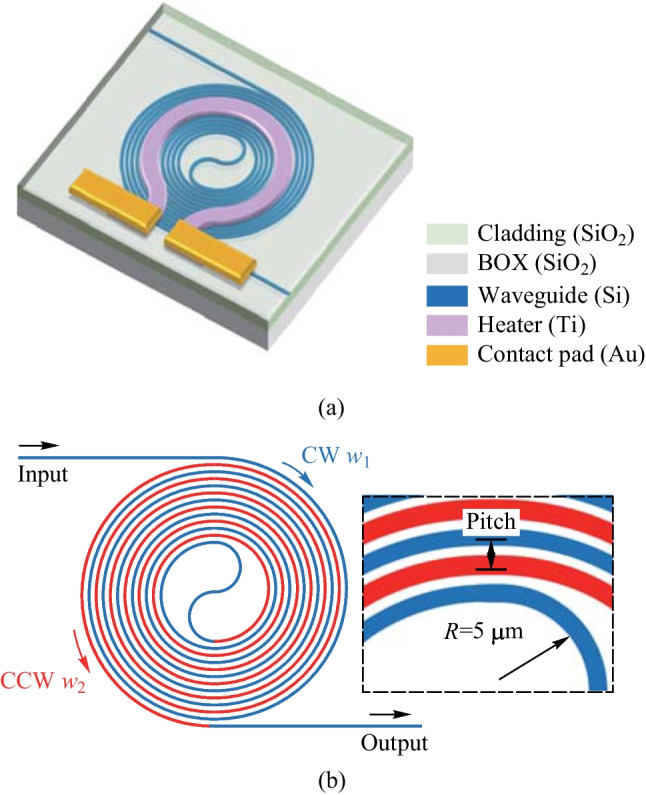
**a** Schematic of the round-spiral phase shifter. **b** Top-view of the round-spiral phase shifter, consisting of CW and CCW spiral waveguides with different widths, i.e., $w_1$ and $w_2$, and an S-bend connector. The insets indicate that the minimum radius is 5 $\upmu {\textrm{m}}$

We have made significant improvements to the design of the racetrack-spiral phase shifter to overcome the drawbacks mentioned earlier. Our new round-spiral phase shifter design not only outperforms the racetrack-spiral design in terms of insertion loss and footprint, but it also features a simpler structure that requires no special design considerations. Additionally, we have achieved nearly complete elimination of reflection losses through optimization. Moreover, we are proud to announce that our new round-spiral phase shifter is fully compatible with standard commercial DUV fabrication processes, representing a major breakthrough in this area. These enhancements have been experimentally validated, and we believe they will have a significant impact on the development of future photonic applications.

**Fig. 13:**
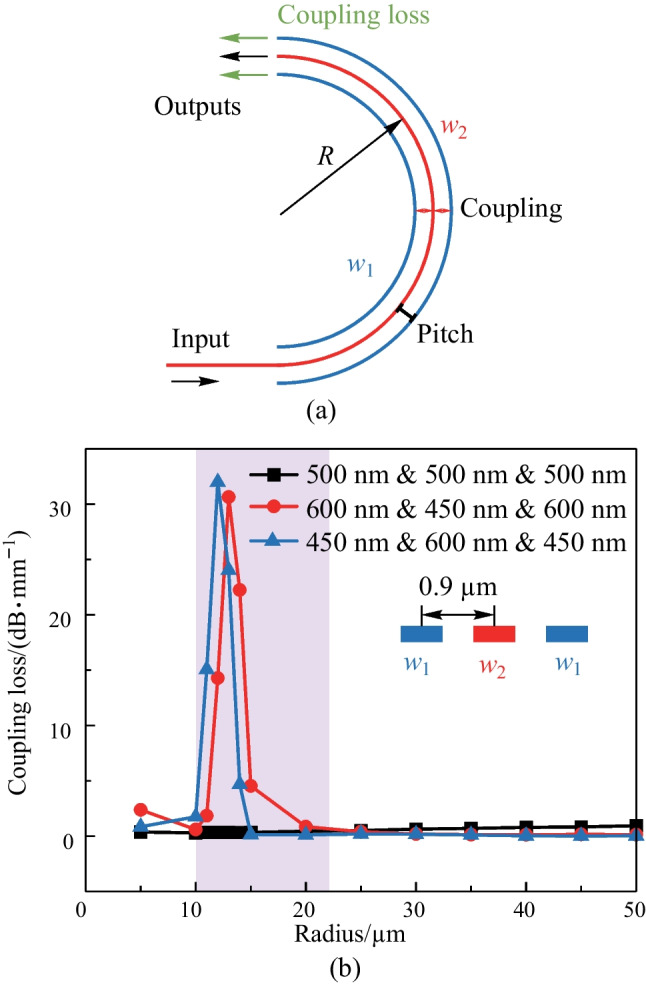
**a** Coupling loss simulation model. **b** Simulated result comparison between different width combinations

In order to reduce power consumption, we have utilized the same principle as the racetrack-spiral phase shifter, which involves the absorption of residual heat by the spiral waveguide. Our proposed round-spiral phase shifter, shown in Fig. 12a, features two round-spiral waveguides with varied widths ($w_1$ and $w_2$) and an S-shaped bend connector, as depicted in the top view of Fig. 12b. Unlike the racetrack-spiral phase shifter, all the waveguides in our design are commonly bent and have no straight waveguide sections. Additionally, the radius of curvature in our design gradually changes, in contrast to the sharp curvature changes present in the racetrack-spiral phase shifter. As a result, the mode mismatch loss is eliminated and offset is unnecessary.

It is worth noting that the minimum radius of the S-bend connector in our design is optimized as 5 $\upmu {\textrm{m}}$, which is significantly larger than the minimum radius of the racetrack-spiral phase shifter (only 1.8 $\upmu {\textrm{m}}$), as indicated in the inset of Fig. 12b. This difference in radius contributes to the reduction of power consumption in our round-spiral phase shifter design, which we have experimentally validated. We believe that these improvements will have a significant impact on the development of future photonic applications.

**Fig. 14:**
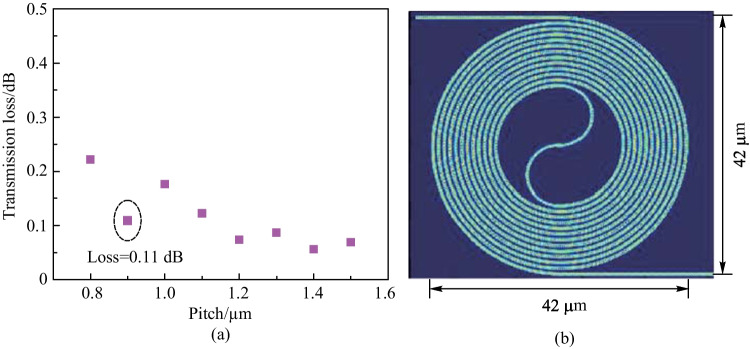
**a** Simulated transmission loss of round-spiral phase shifter with pitch varies from 0.8 to 1.5 $\upmu {\textrm{m}}$ with a step of 0.1 $\upmu {\textrm{m}}$. **b** Simulated light propagation in the phase shifter for $\Lambda$ = 0.9 $\upmu {\textrm{m}}$

We have conducted a simulation and analysis of the coupling loss between the clockwise (CW) and counterclockwise (CCW) spiral waveguides in the round-spiral phase shifter. The study focused on the main coupling loss between adjacent waveguides, as depicted in Fig. 13a. Specifically, the light propagates in the center waveguide with a width of $w_2$ and is coupled to two adjacent waveguides with equal widths of $w_1$ [57]. We set the initial value of the pitch to 0.9 μm. To minimize the coupling loss, we aimed to achieve the mode mismatch among the waveguides. Generally, the mode mismatch is negligible for the straight waveguide due to the equal effective refractive index, resulting in a large coupling loss. Conversely, in sharply bend waveguides, mode mismatch is significant even when the waveguide widths are the same, leading to a smaller coupling loss compared to waveguides with different widths. We simulated the coupling loss for different groups of waveguide widths, and the results are presented in Fig. 13b. We compare the coupling loss for different waveguide width configurations. For the case where $w_1 = w_2 = 500$ nm, the coupling loss remains small compared to the situations where $w_1 = 450$ nm and $w_2 = 600$ nm, or $w_1 = 600$ nm and $w_2 = 450$ nm. In order to determine the appropriate pitch for our design, we varied the pitch and simulate the coupling loss in the overall structure. The simulation result is presented in Fig. 14a. To achieve both low coupling loss and a small footprint, we ultimately selected a pitch of 0.9 μm. Figure 14b illustrates the light propagation in the round-spiral phase shifter with a 0.9-μm pitch.

**Fig. 15:**
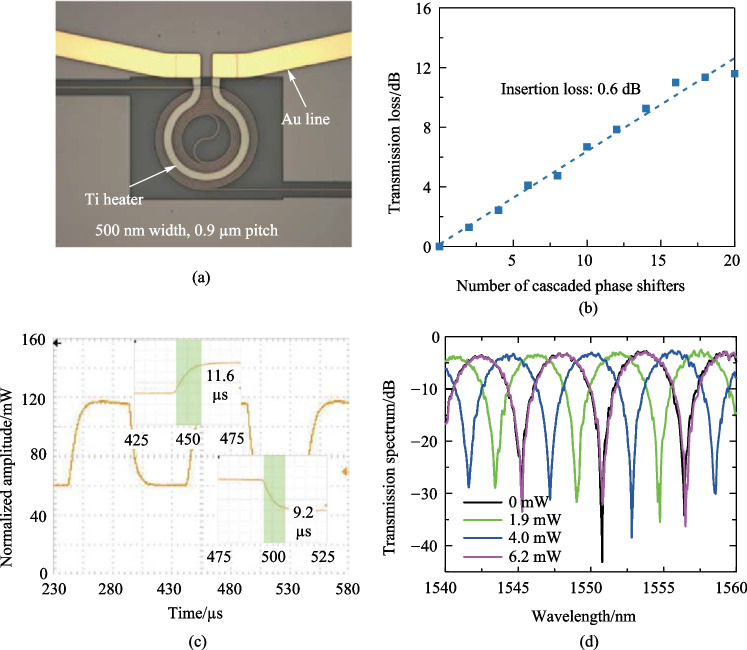
**a** Microscope image of the round-spiral phase shifter. **b** Modulation speed of the round-spiral phase shifter. The rising time and the falling time are 11.6 and 9.2 $\upmu {\textrm{s}}$, respectively, resulting in a modulation speed of 34 kHz. **c** Measured insertion loss of 0.6 dB with the cutback method. **d** Measured power consumption of 3.1 mW/$\uppi$

We utilized the same fabrication process for the round-spiral phase shifter as for the racetrack-spiral phase shifter, as presented in Fig. 15a. Prior to measuring the phase shifter’s performance, we first measured the straight waveguide loss of the 500-nm-width structure using the undercut method and obtained it to be 2.7 dB/cm. We then measured the insertion loss of the round-spiral phase shifter, which was found to be 0.6 dB, as depicted in Fig. 15b. To assess the modulation speed of the 3-$\upmu {\textrm{m}}$ round-spiral heater, we applied a 5 kHz square-wave pulse train and the measured response is displayed in Fig. 15c. The insets of the figure indicated that the rising and falling times were 11.6 $\upmu {\textrm{s}}$ and 9.2 $\upmu {\textrm{s}}$, respectively, enabling us to calculate the cut-off frequency (i.e., modulation speed) as 34 kHz. Finally, we measured the power consumption of the round-spiral phase shifter to be 3.1 mW/$\uppi$, as illustrated in Fig. 15(d).

**Fig. 16:**
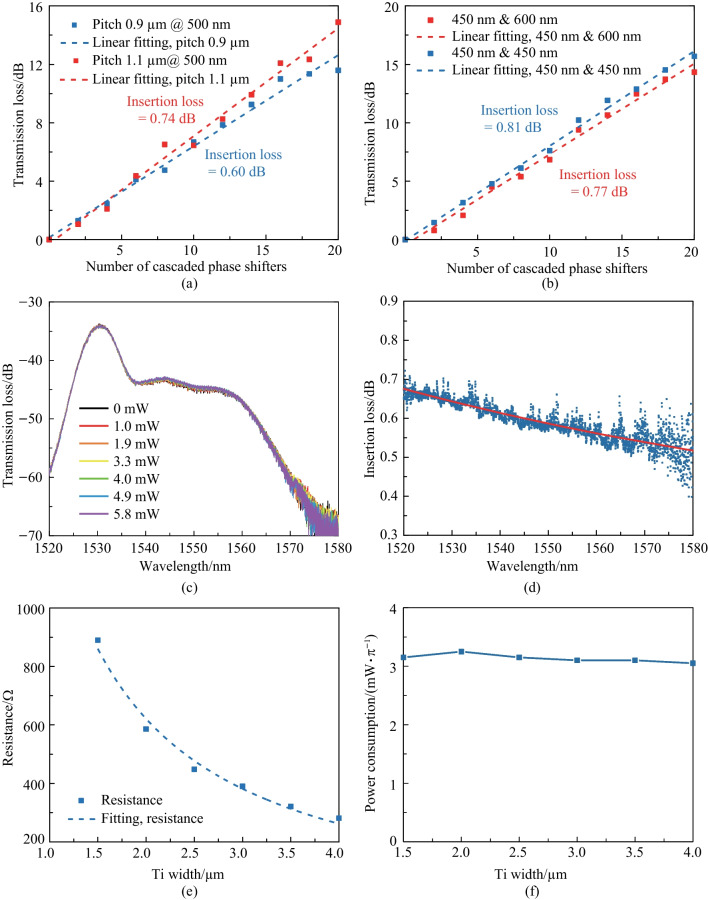
**a** Insertion loss comparison between the pitches of 0.9 and 1.1 $\upmu {\textrm{m}}$, indicating the performance robustness on the pitch. **b** Insertion loss comparison across various width combinations of 450 and 600 nm, indicating the performance robustness on the waveguide width. **c** Negligible power vibration when the phase is changed from 0 to 2$\uppi$. indicating the intensity is independent of the phase. **d** The low insertion loss remains in a large wavelength range, essential for wavelength-tuning-assisted 2D beam steering. **e** Measured resistance of different heater widths. **f** Measured power consumption for different heater widths, indicating the robustness of the heater width

**Table 2:** Comparison of experimental results among silicon phase shifters without the undercut process

Refs.	Fab.	*P*$_\uppi$ /mW	BW ($\tau$) /(kHz ($\upmu {\textrm{s}}$))	Loss /dB	*P*$_\uppi$ $\cdot$ $\tau$/*T* /(mW $\times$ $\upmu {\textrm{s}}$)	Footprint /($\upmu {\textrm{m}}$ $\times$ $\upmu {\textrm{m}}$)
USC 2003 [49]	Standard$^a$	50	100 (3.5)	3.1	357	1200 $\times$ 14
Austin 2007 [50]	Standard	80	18 (20)	5	4919	80 $\times$ 13
Ottawa 2009 [52]	Standard	6.5	25 (14)	3.7	213	130 $\times$ 130
MIT 2013 [45]	Doped Si$^b$	12.7	146 (2.4)	1.7	45	6 $\times$ 6
British Columbia 2015 [53]	Standard	3.8	7.8 (45)	1.2	225	800 $\times$ 180
Columbia 2019 [20]	Standard	1.7	53 (6.6)	4.6	33	850 $\times$ 25
USC 2019 [25]	Standard	2.56	10.1 (35)	1.23	118	109 $\times$ 21
Racetrack-spiral 2020 [56]	Standard	3.0	39 (9)	0.9	33	67 $\times$ 28
HUST 2022 [58]	Side heater$^c$	13.6	186 (1.67)	0.78	28.4	100 $\times$ 2
Round-spiral 2022	Standard	3.1	34 (10.4)	0.6	37	42 $\times$ 42

$^{a}$ Heater deposited on Si waveguide

$^{b}$ Si waveguide doped as a resistor

$^{c}$ Heater deposited besides Si waveguide, not compatible with DUV process in a foundry

Assessing the robustness of a device is a critical factor in determining its suitability for large-scale integration. As previously mentioned, the robustness is a weakness of the racetrack-spiral phase shifter. We conducted a series of robustness tests on the round-spiral phase shifter, focusing on four aspects. Firstly, we ensured that the insertion loss remained small ($<0.8 \text { dB}$) despite large changes in waveguide widths (500 to 450 and 600 nm) or pitch (0.9 to 1.1 $\upmu {\textrm{m}}$), as shown in Fig. 16a, b. This guarantees power consistency in each OPA channel, even in the case of significant fabrication errors. Secondly, we investigated power vibration during phase changes from 0 to 2$\uppi$, and due to the resonant-free waveguide design, we observed negligible power vibration, as shown in Fig. 16c, ensuring OPA stability. Thirdly, we assessed the insertion loss response to the wavelength, demonstrating excellent stability (0.55 to 0.68 dB) in the 1520 to 1580 nm range (limited by the experimental setup), as illustrated in Fig. 16d. This feature is essential for wavelength-based 2D beam steering of common 1D-OPA. Finally, we varied the heater width from 1.5 to 4 $\upmu {\textrm{m}}$, measuring the corresponding power consumption, which remained constant at 3.1 mW/$\uppi$, as shown in Fig. 16e, f.

We compare the proposed two types (racetrack-spiral and round-spiral) of phase shifters with the other thermo-optic phase shifter schemes that have been demonstrated without any undercut process on the silicon platform, and the result is shown in Table 2. There are trade-offs among the performances, such as power consumption, insertion loss, modulation speed (*BW*), and footprint. To evaluate the performance overall aspects, we utilized the figure of merit (FOM) $P_\uppi$ $\cdot$ $\tau$/*T* as the performance metric for the phase shifter [59]. The FOM combines three crucial aspects of a phase shifter: power consumption ($P_\uppi$), modulation speed ($\tau$), and insertion loss (*T*), calculated as $BW = \dfrac{0.35}{\tau }$ based on the relationship between the thermal time constant and modulation speed for RC electric circuits. The transmission coefficient (*T*) is linked to insertion loss (dB unit) by ${\text{Loss}} = \log _{10} T$. Generally, a lower FOM value ($P_\uppi$ $\cdot$ $\tau$/*T*) indicates better overall performance. Compared to other phase shifter schemes, our presented racetrack-spiral and round-spiral phase shifter schemes demonstrate superior performance in terms of low power consumption, low insertion loss, high modulation speed (*BW*), and compact size. The proposed round-spiral phase shifter exhibited the best overall performance.

### Discussion

Optimization of the proposed phase shifter can further improve its performance. By reducing waveguide loss to the state-of-the-art level of $\sim$1 dB/cm and eliminating reflection loss, the insertion loss of the phase shifter could be decreased to 0.4 dB. Moreover, the power consumption of the phase shifter could be reduced to the tens of $\upmu {\textrm{W}}$ level by undercutting the silicon substrate and BOX layer beneath the silicon waveguide, although this comes at the cost of sacrificing the modulation speed of the device. Alternatively, the modulation speed can be significantly improved by utilizing advanced 2-dimensional (2D) materials such as graphene to replace the Ti heater. Therefore, future work can focus on implementing these improvements to further enhance the performance of the phase shifter.

## Energy-efficient optical phased array

### Theory of periodic OPA

**Fig. 17:**
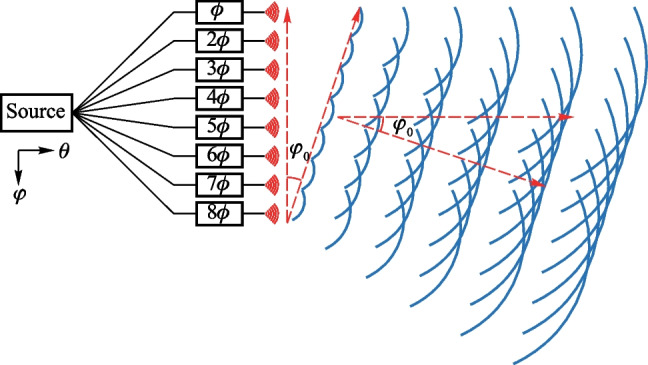
Principle of uniform 1D-OPA

The double-slit experiment, also known as Young’s interference experiment, is widely recognized as an instance of double-beam interference. Building on this concept, the fundamental principle underlying the OPA is that of multi-beam interference in the far field. The operational principle of the periodic one-dimensional (1D) OPA is depicted in Fig. 17. In this system, the $\varphi$ direction is defined as parallel to the emitter distribution direction, while the direction perpendicular to it is termed the $\theta$ direction. Typically, the light produced by the source is segregated into several channels, as depicted by the eight channels in the figure. Each channel is modulated with a unique phase, with a fixed phase difference of $\phi$ between adjacent channels. Benefiting from this, the light from each channel is emitted into free space and the wavefront direction is inclined at an angle of $\varphi _0$ against the horizontal line. In keeping with Young’s interference experiment, we consider the first emitter with a phase of $\phi$ as the reference or origin. Furthermore, we set the amplitude of each channel to a simplified value of “1” (uniform power distribution), and the phase of *n*th channel can be written as $\phi _n=e^{j \frac{2\uppi }{\lambda } x_n \sin {\varphi _0}}$. Then the far-field $E(\varphi )$ can be written as

2 $$\begin{aligned} E(\varphi ) = \sum _{n=1}^N e^{j \frac{2\uppi }{\lambda } x_n (\sin {\varphi }-\sin {\varphi _0})} ,\end{aligned}$$

where $x_n$ represents the distance between the *n*th channel and the the first channel (origin). By utilizing the Euler equation and Trigonometric formulas, The normalized far-field power distribution, which is depicted from Eq. (2), can be expressed as

3 $$\begin{aligned} P(\varphi ) = \frac{1}{N^2} \cdot \left| \frac{\sin {\left[ \frac{N\uppi d}{\lambda }(\sin {\varphi }-\sin {\varphi _0})\right] }}{\sin {\left[ \frac{\uppi d}{\lambda }(\sin {\varphi }-\sin {\varphi _0})\right] }} \right| ^2 .\end{aligned}$$

**Fig. 18:**
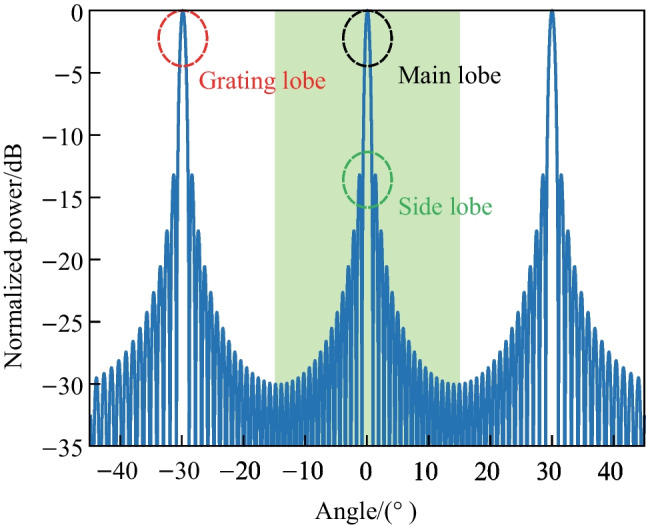
Far-field power distribution of the condition as $\frac{d}{\lambda } = 2$

The power distribution, $P(\varphi )$, in the far field of the OPA is predominantly determined by $\frac{d}{\lambda }$. For instance, by setting $\frac{d}{\lambda } = 2$, the far field figure is illustrated in Fig. 18. Typically, the primary peak at $\varphi _0$ (0$^{\circ }$ in Fig. 18) is recognized as the “main lobe”, while the additional peaks are designated as the “grating lobes”. The power in the grating lobes is regarded as wasteful and leads to significant insertion loss. Generally, only the power in the main lobe is useful for practical applications, such as in Lidar. The presence of numerous “small peaks” outside the main lobe, referred to as “side lobes” and illustrated in the green circle, have a crucial role in determining the signal-to-noise ratio in the far field. The quality of the 3D point cloud may be affected by the presence of side lobes.

**Fig. 19:**
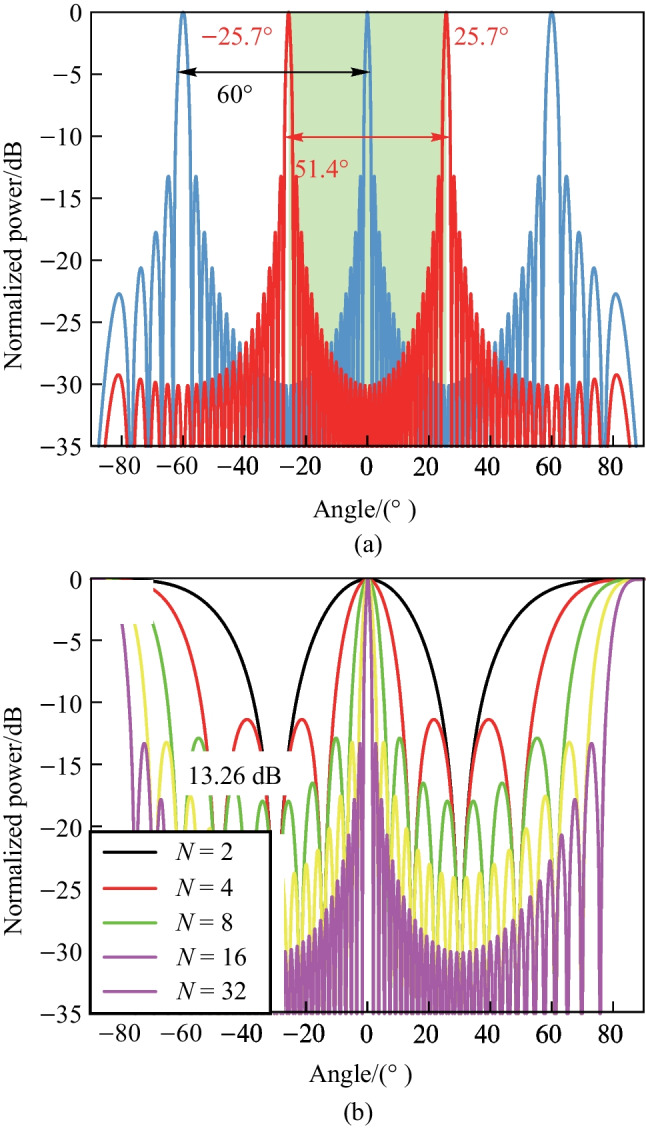
**a** Right FoV illustration of $\frac{d}{\lambda }=\frac{2}{\sqrt{3}}$, blue line: $\varphi _0$ = 0$^{\circ }$, red line: $\varphi _0$ = 25.7$^{\circ }$. **b** Far-field power distribution varies *N*. The limitation for SLSR is theoretically calculated as 13.26 dB for periodic OPA with uniform power distribution

There are some metrics for describing the performance of an OPA, such as field of view (FoV) and sidelobe suppression ratio (SLSR). The FoV is the extent of the observable world that is seen at any given moment. FoV refers to the maximum steering range where only the main lobe exists, with no other grating lobe present. Typically, the FoV is symmetrical with respect to $\varphi$ = 0$^{\circ }$. When the grating lobe condition, represented by $\frac{\uppi d}{\lambda } (\sin {\varphi }-\sin {\varphi _0}) = 0, \pm \uppi , \pm 2\uppi , \pm 3\uppi ...$, is satisfied, the grating lobe will appear, the formula can be further expressed as

4 $$\begin{aligned} \frac{d}{\lambda } = \frac{n}{\sin {\varphi _{\text{GL}}}-\sin {\varphi _0}}. \end{aligned}$$

It is important to emphasize that the correct method for calculating the FoV is to assume $\varphi _0 = -\varphi _\text{GL}$ rather than setting $\varphi _0 = 0$ This is a common error made by researchers. The condition of $\varphi _0 = -\varphi _\text{GL}$ indicates the angle at which the grating lobe appears when the beam is steered to $-\varphi _\text{GL}$, and the FoV will be equal to 2$\varphi _\text{GL}$. For instance, the correct FoV for $\frac{d}{\lambda }=\frac{2}{\sqrt{3}}$ is 51.4$^{\circ }$, instead of 60$^{\circ }$, as illustrated in Fig. 19a. Then the FoV equation can be expressed as

5 $$\begin{aligned} \frac{d}{\lambda }=\frac{1}{2 \sin {\left( \frac{\text{FoV}}{2}\right) }}. \end{aligned}$$

The side lobe refers to the small peaks located between two adjacent grating lobes. As previously mentioned, the far field figure of 1D OPA which has uniform power distribution can be represented by a *Sinc* function, as Eq. (3) expresses. The solutions of this function correspond to the peaks between the grating lobes. We can calculate the derivative and determine the conditions for the occurrence of these peaks as follows:

6 $$\begin{aligned} \left| E(\varphi ) \right| = \frac{1}{N} \cdot \left| \frac{\sin {\left( 1.4303 \uppi \right) }}{\sin {\left( \frac{1.4303 \uppi }{N}\right) }} \right|. \end{aligned}$$

For large-scale OPAs, when the number of elements *N* is sufficiently large, the highest side lobe, which is also the closest side lobe to the main lobe, is located at an angular position close to that of the main lobe, denoted as $\varphi _0$. In this case, the limitation of the SLSR can be calculated as 13.26 dB with ease, which is shown in Fig. 19b. A comprehensible way to interpret this phenomenon is to consider the *Sinc*-function-shaped far field pattern as the Fourier transform of the gate function, which represents the uniform power distribution of the OPA in the near field.

### Energy-efficient and high SLSR periodic OPA

**Fig. 20:**
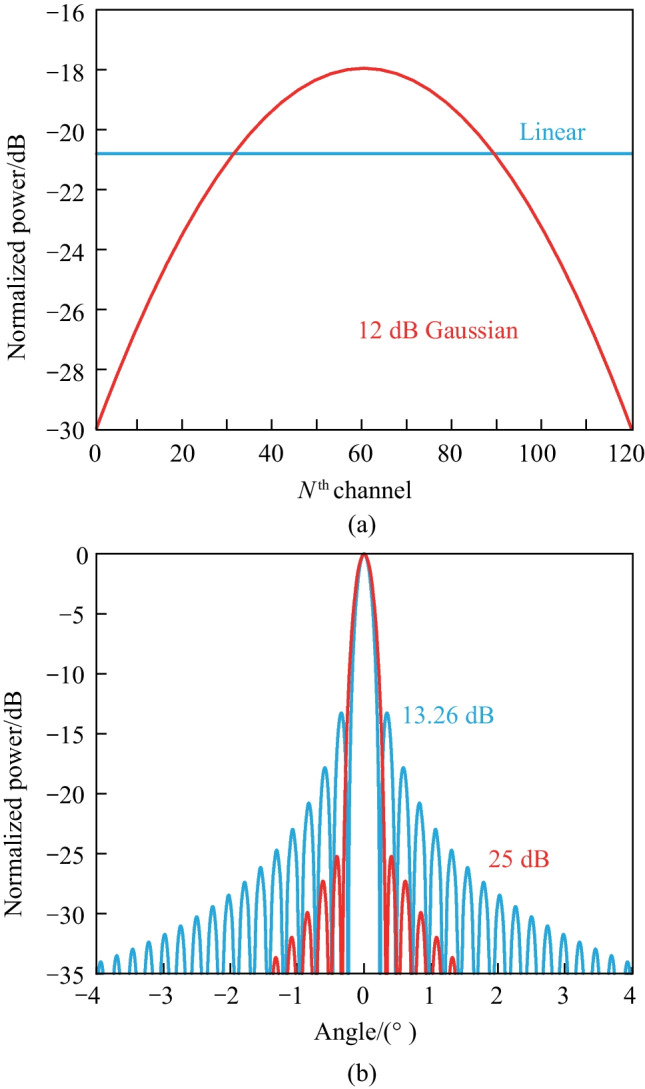
Near and far fields comparison between linear and Gaussian power distribution, blue: uniform, red: Gaussian. **a** The uniform and Gaussian power distribution in the near field. **b** The corresponding far-field power distribution, 12-dB Gaussian power distribution represents 25 dB SLSR

For OPA-based applications, particularly for free-space optical communication, a higher SLSR implies better signal quality. As a result, the need to increase the SLSR of integrated OPA has become crucial. The 13.2-dB-SLSR limitation mainly arises from the Fourier transform of the gate function. It is widely recognized that the Fourier transform of a Gaussian function is also a Gaussian function. Therefore, the Gaussian envelope in the near-field can contribute to the improvement of SLSR. To demonstrate this, we compare the near-field and far-field figures between uniform and Gaussian power distributions with a spacing of $\frac{d}{\lambda } = 2$ and *N* channels. In Fig. 20a, b, the blue and red curves represent the uniform and Gaussian power distributions, respectively. We set the center-to-edge of the Gaussian power distribution to be 12 dB in the near-field, resulting in a significant improvement in the corresponding SLSR of 25 dB in the far field. However, one drawback of the method is that achieving a high SLSR comes at the expense of a broader beamwidth, although it is negligible in Fig. 20b. This is because the effective emitting area is smaller for the Gaussian power distribution than for the uniform power distribution.

**Fig. 21:**
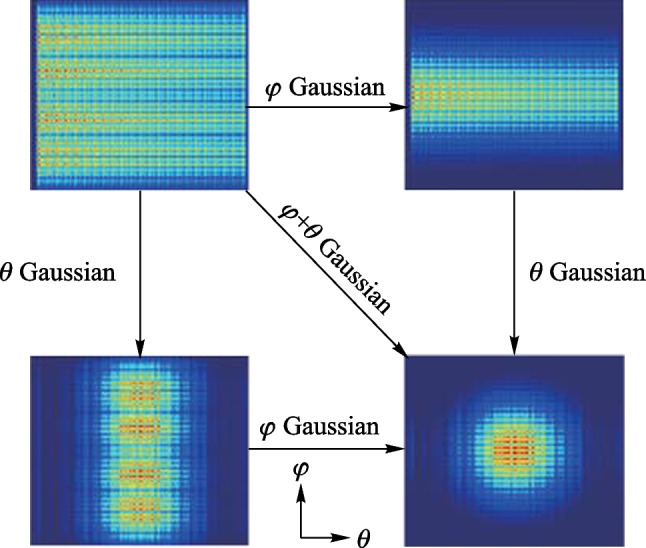
Near-fields simulation of linear, $\varphi$ Gaussian, $\theta$ Gaussian, and $\varphi$ & $\theta$ Gaussian power distribution

Several schemes have been proposed to generate a Gaussian power distribution in the $\varphi$ direction by employing a star coupler [41, 46, 60, 61]. However, limited research has focused on achieving such a distribution in the $\theta$ direction. In this work, we present a design that enables generating a Gaussian power distribution in both the $\varphi$ and $\theta$ directions, which in turn yields an ideal round-Gaussian pattern in the far field. To verify our approach, we have designed a 16-channel 1D-OPA with a pitch of 2$\lambda$ (i.e., 3.1 $\upmu {\textrm{m}}$) and employed ANSYS’s Lumerical FDTD to simulate its near-field figure. Specifically, we have separately simulated the linear power distribution, $\varphi$ Gaussian power distribution, $\theta$ Gaussian power distribution, and $\varphi$ & $\theta$ Gaussian power distribution, and the corresponding near-field figures are presented in Fig. 21. Our results demonstrate that, compared to the linear power distribution, the $\varphi$ & $\theta$ Gaussian power distribution generates a Gaussian function in the near field, indicating a Gaussian power distribution in the far field via Fourier transform.

In the $\varphi$ direction, a commonly employed method to achieve a Gaussian power distribution is through the use of a star coupler, as mentioned before. This approach enables the shortest path for splitting light and produces a perfect Gaussian power divider due to its specifically designed structure. However, the design becomes more challenging and sensitive to fabrication errors as the number of channels, *N*, increases. Additionally, the star coupler is limited to generating only Gaussian power distributions and cannot produce other power distributions.

**Fig. 22:**
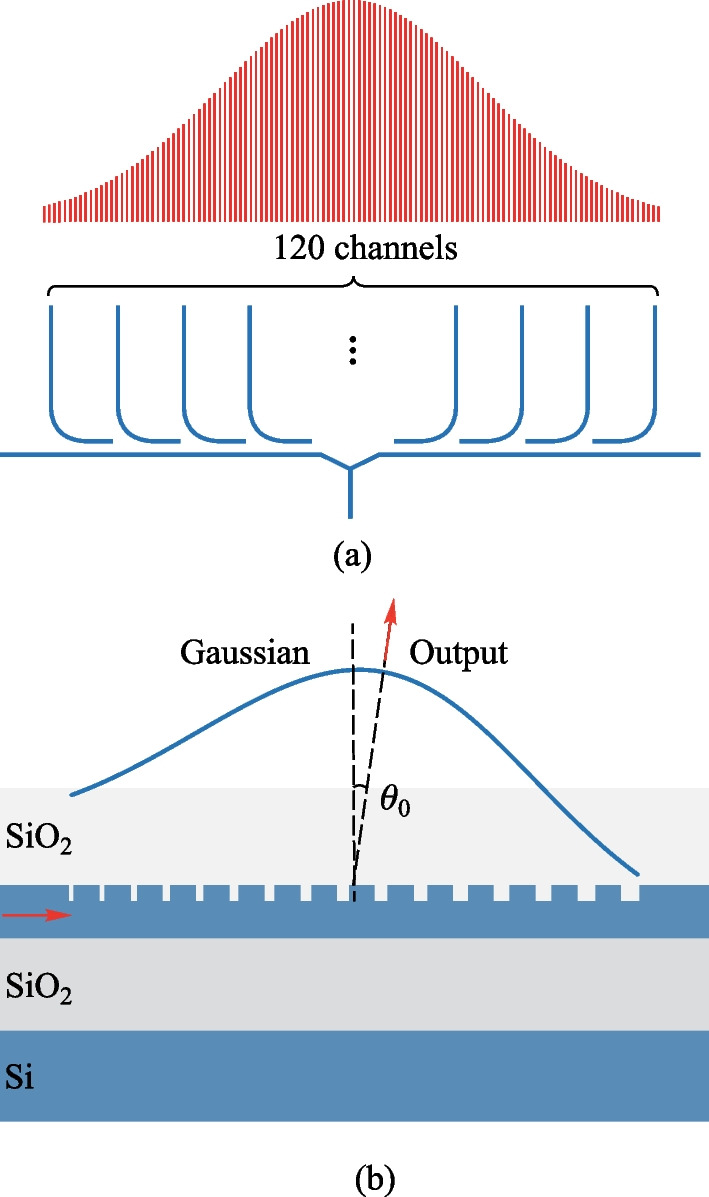
**a** Schematic of the Y-splitter-assisted cascaded coupler to realize the Gaussian power distribution in the $\varphi$ direction. **b** Schematic of the apodized grating emitter to achieve Gaussian power distribution in the $\theta$ direction

To enable more flexible and easily designed power adjustments, we employ a Y-splitter-assisted cascaded coupler to distribute the power and form the desired Gaussian distribution, as depicted in Fig. 22a. As the power required at the center of the Gaussian power distribution is much larger than that at the edge, the utilization of a Y-splitter allows light to be coupled from the center to the edge. This approach significantly broadens the range of the number of channels that can be supported and the center-to-edge ratio of the resulting Gaussian function. In addition, we have designed an apodized grating emitter to achieve the Gaussian power distribution in the $\theta$ direction, as illustrated in Fig. 22b. The pitch and duty cycle of the grating emitter determine the emitting angle in the far field, and the efficiency of each grating unit is solely dependent on the duty cycle when the etch depth is fixed. To build a 2-mm-long emitting grating and ensure a small beamwidth or spatial resolution in the $\theta$ direction, we have chosen a shallow etch depth of 10 nm. Furthermore, the shallow etching has the added benefit of minimizing the effect of duty cycle changes on the effective refractive index, i.e., the emitting angle of the grating. We adjust the duty cycle of each unit to form the Gaussian power distribution along the grating. To ensure that the emitting angle of each unit is consistent, we slightly adjust the pitch of each unit (with a maximum adjustment of only 4 nm) [62, 63]. Finally, we design 12-dB Gaussian power distribution in both $\varphi$ and $\theta$ directions (dual-Gaussian) to obtain the 25 dB SLSR in the far field.

**Fig. 23:**
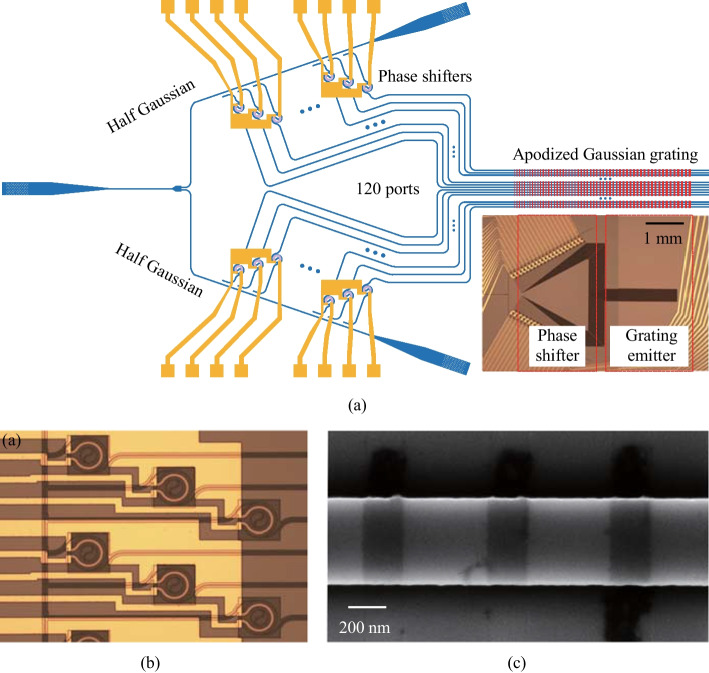
**a** Schematic of proposed periodic 1D-OPA, inset: microscope image of the fabricated OPA. **b** Zoomed-in microscope image of the round-spiral phase shifters. **c** SEM image of the shallow-etched apodized grating emitter

**Fig. 24:**
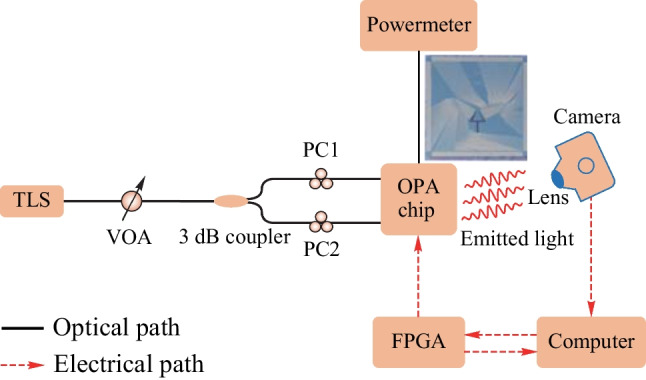
Experimental setup of OPA

**Fig. 25:**
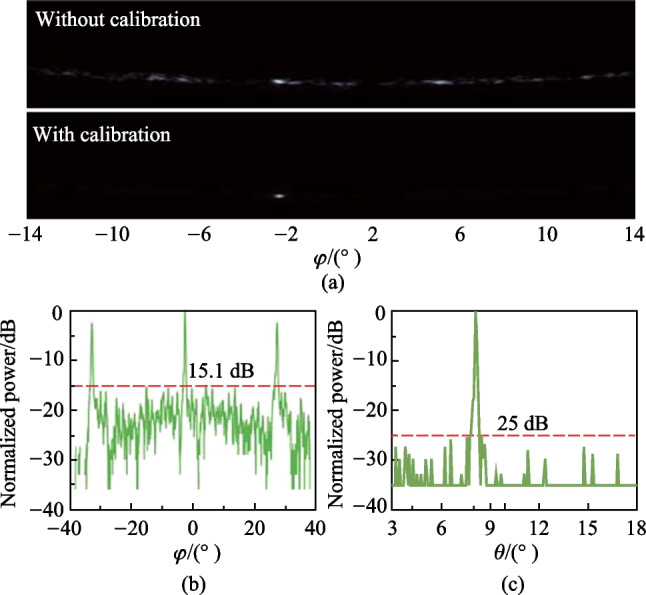
**a** The far-field figure before and after calibration. **b** Cross-section in the $\varphi$ direction, the SLSR is 15.1 dB at $\varphi$ = $-$2.4$^{\circ }$ for the maximum noise obtained. **c** Cross-section in the $\theta$ whose SLSR is 25 dB

The designed 120-channel dual-Gaussian 1D-OPA is illustrated in Fig. 23a, the pitch between the waveguides is 3.1 $\upmu {\textrm{m}}$, equal to 2$\lambda$. The inset shows the microscope image of the fabricated OPA. The input light is coupled from the fiber to the waveguide using a vertical grating coupler, following which the light is split equally into two parts and transmitted in opposite directions. A Y-splitter-assisted cascaded coupler is employed to achieve the 12 dB Gaussian power distribution in the $\varphi$ direction. The phase of each channel is modulated individually by a round-spiral phase shifter and then transmitted to the apodized grating emitter, which achieves the 12 dB Gaussian power distribution in the $\theta$ direction. The optical path of each channel is designed uniformly, which facilitates far-field calibration. All the phase shifters are controlled by the electrical signals present on I/O pads. Due to the low 2$\uppi$ driving voltage (1.4 V) of the energy-efficient phase shifter, an electrical signal generated by a commercial FPGA can directly drive it. This feature provides significant benefits for system integration and reduces complexity in the design of the control circuitry. Moreover, every three-phase shifters share a common ground pad (GND) limited by the FPGA. Figure 23b presents a zoomed-in image of the energy-efficient round-spiral phase shifter, where the average resistance measures 536 $\Omega$. We have implemented the stepped distribution of every three-phase-shifter with a horizontal pitch of 100 $\upmu {\textrm{m}}$ and a vertical pitch of 40 $\upmu {\textrm{m}}$ to reduce thermal crosstalk. The shallow-etched grating is displayed in the SEM image of Fig. 23c.

**Table 3:** Performance comparison among state-of-art periodic 1D-OPAs

Year	2018 [64]	2020 [20]	2021 [65]	2022$^a$ [46]	2023 (This work)
Platform	Si	Si	$\mathrm {Si_3N_4/Si}$	Si	Si
Number of channels	1024	512	64	64	120
FoV ($\varphi$ $\times$ $\theta$)	40$^{\circ }$ ($\varphi$)	70$^{\circ }$ $\times$ 6$^{\circ }$	35.5$^{\circ }$ $\times$ 22.7$^{\circ }$	140$^{\circ }$ $\times$ 13.5$^{\circ }$	25$^{\circ }$ $\times$ 13.2$^{\circ }$
SLSR/dB	9 ($\varphi$)	9 ($\varphi$)	5 ($\varphi$)	19 ($\varphi$)	15.1 $\times$ 25
Aperture/mm	2 ($\varphi$)	2 $\times$ 1.6	0.16 $\times$ 2	0.05 $\times$ 2	0.363 $\times$ 2
Beamwidth ($\varphi$)	0.03$^{\circ }$ ($\varphi$)	0.15$^{\circ }$ $\times$ 0.08$^{\circ }$	0.69$^{\circ }$ $\times$ 0.075$^{\circ }$	2$^{\circ }$ $\times$ 0.07$^{\circ }$	0.31$^{\circ }$ $\times$ 0.07$^{\circ }$
$P_\uppi$	54	1.7	17.5 (Si)	7.1	2.7

$^{a}$ Gaussian power distribution realized by star coupler

The experimental setup, as illustrated in Fig. 24, A tunable laser source (TLS) generates a laser beam of constant power, which is then adjusted by a discrete variable optical attenuator (VOA) before being coupled to the OPA chip. The light is split into two beams by a 3 dB coupler and is individually controlled by polarization controllers (PCs). One beam is utilized for real-time monitoring of the coupling status, and its output is connected to a power meter. The other beam is emitted to the far field after passing through the OPA chip and is captured by an infrared camera. The chip is controlled by a commercial FPGA, and a Python script running on a computer analyzes the received image from the infrared camera to optimize the far-field intensity of the target angle and achieve the best performance. The black solid lines denote the optical path, while the red dashed arrows represent the electrical path. The output voltage is generally fixed at 3.3 V and is not adjustable for commercial FPGA. We employ pulse width modulation (PWM) to vary the output voltage and adjust the phase of the phase shifters. Additionally, we utilize the gradient descent algorithm to optimize each of the 120 phase shifters individually by the captured far-field information of the infrared camera.

We set the wavelength to 1550 nm and present the far-field pattern before calibration in Fig. 25a. Due to the equal-optical-path design for each channel, there exists a relatively bright spot in the far field, even though the phase shifters are not calibrated. The brightest spot is selected and optimized using the gradient descent algorithm. After calibration, the power is more concentrated. The cross-section in the $\varphi$ direction is shown in Fig. 25b, which is obtained by collecting the maximum power at each $\varphi$ value. The maximum noise, i.e., the sidelobe power, is thus obtained, and the FoV and the $\varphi$ SLSR are found to be 29$^{\circ }$ and 15.1 dB, respectively. The measured $\varphi$ SLSR of 15.1 dB differs from the design value of 25 dB mainly due to the noise of the PWM signal and the common-ground scheme, which are limited by the quality of the commercial FPGA. The cross-section in the $\theta$ direction is shown in Fig. 25c, and the measured $\theta$ SLSR is 25 dB, which agrees well with the theoretical value of 25 dB.

**Fig. 26:**
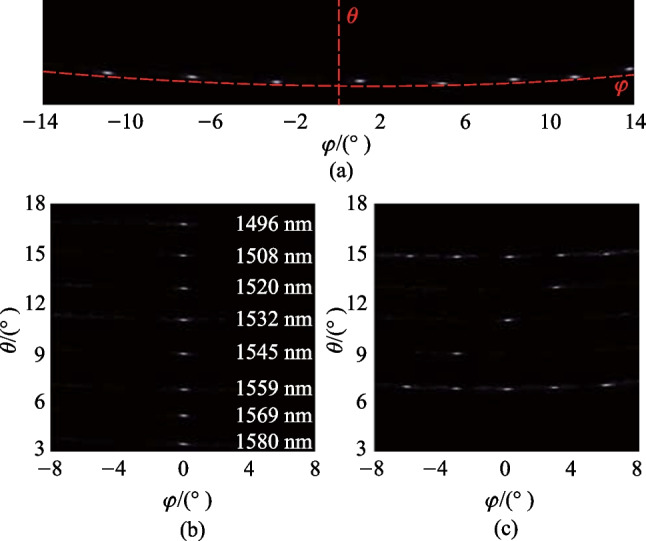
**a** Beam steering in the $\varphi$ direction from – 11$^{\circ }$ to 14$^{\circ }$ within the FoV. **b** Beam steering in the $\varphi$ direction by the wavelength tuning. **c** 2D beam steering demonstration to form the character “Z”

We tune the 120 phase shifters to achieve beam steering within an FoV range in the $\varphi$ direction, as shown in Fig. 26a. The beam is steered from – 11$^{\circ }$ to 14$^{\circ }$. Then, we adjust the wavelength to achieve beam steering in the $\theta$ direction, as shown in Fig. 26b. At a wavelength of 1550 nm, the $\theta$ is 8$^{\circ }$, and we steer the beam from 16.7$^{\circ }$ to 3.5$^{\circ }$ by increasing the wavelength from 1496 nm to 1580 nm. The tuning efficiency is   0.16$^{\circ }$/nm. Finally, by simultaneously tuning the wavelength and phase shifters, we 2D-steer the beam to form a character “Z”, as illustrated in Fig. 26c.

**Fig. 27:**
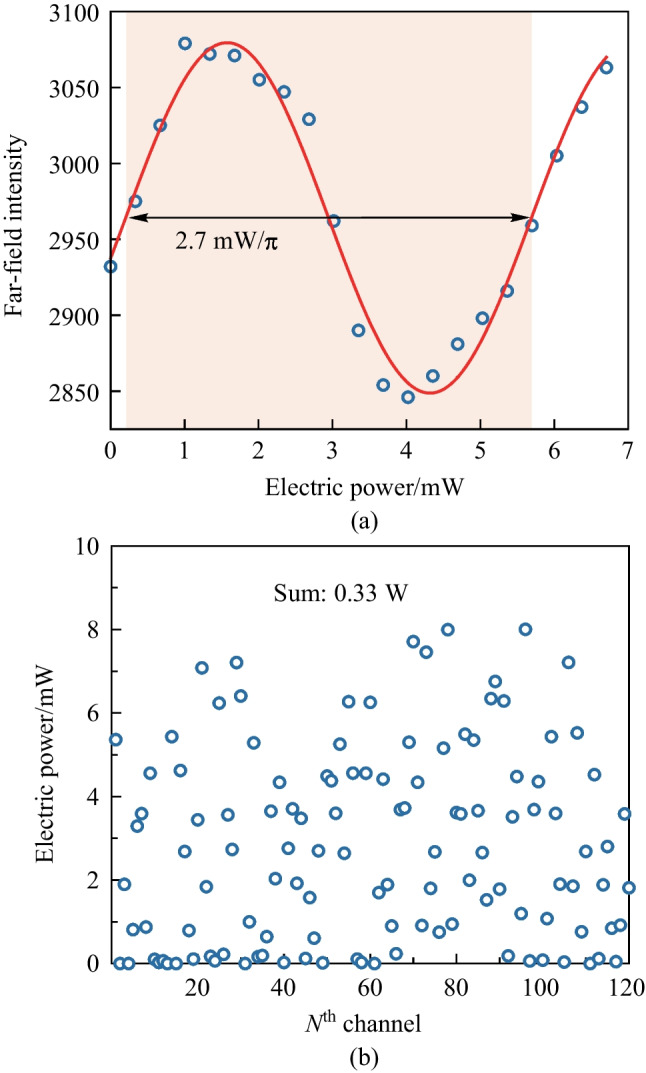
**a** The far-field intensity change when electric power increases from 0 to 7 mW, indicating the power consumption of 2.7 mW/$\uppi$. **b** The maximum total power consumption is 0.33 W when the beam is steered within the FoV

After calibrating the far-field beam, we vary the electric power on a specific channel and obtain the far-field intensity values. The far-field intensity versus electric power relationship is shown in Fig. 27a. The experimental data (blue circle) is fitted by *Sin*-function (solid red curve), and the calculated power consumption of the round-spiral phase shifter is 2.7 mW/$\uppi$, which matches well with the result of the individually measured phase shifter. After that, we steer the beam to the largest angle within the FoV, i.e., 14$^{\circ }$ for the 3.1-$\upmu {\textrm{m}}$-pitch OPA and obtain the power consumption for each channel, the result is shown in Fig. 27b, indicating a maximum power consumption of only 0.33 W.

### Discussion

We conducted a comparative analysis of our proposed energy-efficient OPA with other state-of-the-art thermo-optic-based OPAs, and the results are presented in Table 3. Our scheme demonstrated a significant reduction in thermo-optic power consumption (2.7 mW/$\uppi$) when compared to other existing OPA schemes. Additionally, our proposed scheme exhibited a larger SLSR in both directions when compared to the other schemes.

## Conclusion

In order to decrease power consumption while keeping a good balance on other aspects such as modulation speed, insertion loss, and footprint, we experimentally proposed and demonstrated two energy-efficient phase shifter schemes, i.e., the racetrack-spiral phase shifter and the round-spiral phase shifter. Although the racetrack-spiral phase shifter performs well on all aspects mentioned above, the structure can still be improved further to be fully compatible with the standard commercial DUV fabrication process. After improvement, the round-spiral phase shifter achieved a well-balanced performance on power consumption (3.1 mW/$\uppi$), modulation speed (34 kHz), insertion loss (0.6 dB), and footprint (42 $\upmu {\textrm{m}}$ $\times$ 42 $\upmu {\textrm{m}}$). Moreover, the phase shifter is fabrication-robust to waveguide width, pitch, and heater width. It can be packaged as a building block and is pretty suitable for large-scale integration.

Using the round-spiral phase shifter, we have experimentally demonstrated an optical phased array with excellent energy efficiency. Specifically, the power consumption for each channel was measured to be only 2.7 mW/$\uppi$, and the maximum total power consumption during beam steering within the field of view was only 0.33 W. In addition, we applied a Gaussian power distribution to both the $\varphi$ and $\theta$ directions and achieved impressive sidelobe suppression ratios of 15.1 and 25 dB in the $\varphi$ and $\theta$ directions, respectively.

## Data Availability

The data that support the findings of this study are available from the corresponding author, upon reasonable request.
